# The domain interface method: a general-purpose non-intrusive technique for non-conforming domain decomposition problems

**DOI:** 10.1007/s00466-015-1239-x

**Published:** 2015-12-28

**Authors:** M. Cafiero, O. Lloberas-Valls, J. Cante, J. Oliver

**Affiliations:** CIMNE – Centre Internacional de Metodes Numerics en Enginyeria, Barcelona, Spain; E.T.S d’Enginyers de Camins, Canals i Ports, Technical University of Catalonia (Barcelona Tech), Campus Nord UPC, Mòdul C-1, c/ Jordi Girona 1-3, 08034 Barcelona, Spain; E.T.S. d’Enginyeries Industrial i Aeronàutica de Terrassa, Technical University of Catalonia (Barcelona Tech), Campus Terrassa UPC, c/ Colom 11, 08222 Terrassa, Spain

**Keywords:** Domain decomposition methods, Non-conforming interface, Weak coupling techniques for non-matching meshes

## Abstract

A domain decomposition technique is proposed which is capable of properly connecting arbitrary non-conforming interfaces. The strategy essentially consists in considering a fictitious zero-width interface between the non-matching meshes which is discretized using a Delaunay triangulation. Continuity is satisfied across domains through normal and tangential stresses provided by the discretized interface and inserted in the formulation in the form of Lagrange multipliers. The final structure of the global system of equations resembles the dual assembly of substructures where the Lagrange multipliers are employed to nullify the gap between domains. A new approach to handle floating subdomains is outlined which can be implemented without significantly altering the structure of standard industrial finite element codes. The effectiveness of the developed algorithm is demonstrated through a patch test example and a number of tests that highlight the accuracy of the methodology and independence of the results with respect to the framework parameters. Considering its high degree of flexibility and non-intrusive character, the proposed domain decomposition framework is regarded as an attractive alternative to other established techniques such as the mortar approach.

## Introduction

Modern engineering applications require sophisticated simulation techniques which deal with increasing complexity and refinement of the computational models. Consequently, detailed finite element discretizations are commonly used in nowadays structural analysis and a number of practical situations are emerging in which special techniques are indispensable to handle non-matching discretizations. In this introduction we focus on engineering applications and computational techniques concerning the assembly and resolution of models involving non-overlapping meshes.

### The need for non-matching mesh assemblies in computational mechanics

Typical scenarios arise when independent mesh discretizations are applied to different parts of a structure and when large models are divided and distributed among different working teams. A common situation is encountered when particular structural components are reused in evolving designs such as the wings among diverse aircraft models with changing fuselages. The meshes of the structural components are most likely non-matching and need to be assembled along common edges using special techniques that account for the non-conforming interfaces [34, 35].

The field of contact mechanics [51] has significantly boosted the formulation of new assembly techniques since the most general situations between contact surfaces are encountered therein, e.g. sliding bodies over a surface or rolling and rebounding of different discretized entities. Other somehow related applications are connected with fluid structure interaction [7] and multiphysics [14, 41] analysis where different discretizations are taken into account due to the distinct physical nature of interacting components.

An emerging set of techniques intimately related with computational material design are the so-called multiscale and multiresolution methods [13, 17, 27]. The idea is to account for the lower scale components and their interactions with an upper scale level. Multiresolution techniques based on mesh adaptivity [33] and concurrent multiscale analysis such as global/local approaches [8], variational multiscale methods [25, 26, 36] and multiscale domain decomposition methods [15, 20, 28] are examples in which lower (fine) scale discretizations are glued to upper (coarse) scale models. This can be performed selectively during the computations at areas of interest, e.g. stress concentrations, crack growth and appearance of non-linear effects, by “zooming” into these regions and substituting a part of the domain discretization by its corresponding refined model [30, 31]. As a result, a number of non-conforming interfaces between different scale discretizations arise which need to be handled by appropriate techniques.

Most of the above mentioned applications involve complex models which upon discretization, e.g. using finite elements, lead to large systems of equations. It is then not surprising that most of the existing methodologies to connect different meshes are frequently encountered in domain decomposition techniques [19]. These techniques roughly divide the complex model into subdomains and distribute the corresponding calculations among different processor units. They can be viewed as powerful parallel solvers typically formed by a blend of direct solvers that account for the local domain factorizations and iterative solvers for an interface problem that accounts for the connectivity of all domains. It is precisely in the generation of such connectivity where the techniques discussed in this paper play a crucial role since they ensure continuity of the solution field across all conforming and non-conforming interfaces.

### Non-overlapping domain decomposition analysis with non-conforming interfaces

**Fig. 1:**
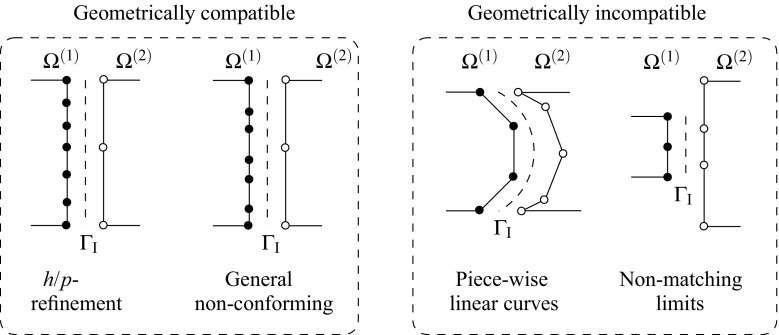
Different situations for a non-conforming interface

In most of the domain decomposition applications the geometrical compatibility of the interface $\Gamma _{\text {I}}$ is assumed to be satisfied between non-matching meshes [44] meaning that the boundaries of the domains at the common interface are identical in the undeformed configuration. However, there are practical situations in which this is not the case, e.g. curved interfaces with different piece-wise linear discretizations (cf. Fig. 1). Extensions to geometrical incompatibility have been specially addressed by contact domain techniques [43, 51]. Domain decomposition techniques designed to tackle non-conforming interfaces typically employ strong or weak coupling constraints to satisfy compatibility of the solution field at the interface.

Strong coupling constraints refer essentially to collocation techniques where one constraint is assigned to each degree of freedom (DOF) while weak coupling techniques refer to constraints enforced in an integral or average sense along portions of the interface surface assigning one constraint per group of DOFs belonging to the interface portion. In the contact domain literature these techniques are often referred to as node-to-segment techniques and segment-to-segment techniques, respectively.

**Fig. 2:**
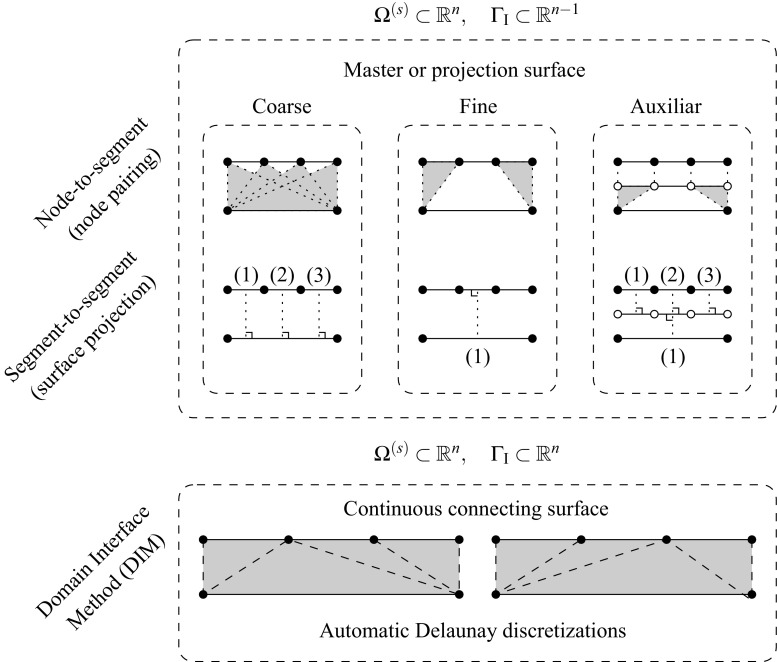
Connecting non-conforming interfaces with different strategies

Generally, a reference displacement field ${\mathbf{u}}_{\Gamma _{\text {I}}}$ is outlined from which the interface constraints are generated. Such field can be chosen based on one of the domain discretizations at the common interface or by a third auxiliary interface discretization with a particular optimal distribution of the DOFs such that the gap between domains is minimized in some norm (cf. Fig. 2). Note that if the coarsest discretization is utilized for the reference solution field the solution can be artificially stiffened if the fine mesh is not obtained by *h* or *p* refinement of the coarse mesh at the interface for the strong coupling case. For this reason, weak coupling techniques are preferred in the context of a general non-conforming interface since they relax the constraint such that stiffening and locking effects do not occur. Mortar methods and their formulation in terms of finite elements constitute the most general technique for non-conforming interfaces in which geometrical incompatibilities can be handled [5]. The most challenging applications of such techniques are identified in time-dependent domains, e.g. contact problems, and when highly refined domains are considered at local areas without the need for an expensive mesh adaption, e.g. multiresolution analysis.

The enforcement of constraints at a non-conforming interface is typically done with the introduction of Lagrange multipliers [1]. Basically, an extra term is added into the variational statement which corresponds to the work performed at the non-conforming interface in terms of the existing gap and interface stresses. In the context of the finite element method different discretizations are generated at each domain and distribution functions are associated with the Lagrange multipliers at the interface leading to a discretized weak form of the equilibrium equations and the interface compatibility constraints. The distribution functions for the Lagrange multipliers and shape functions for the finite elements should be properly selected to fulfill the Ladyzhenskaya–Babuška–Brezzi (LBB) condition (also known as the *inf-sup* condition) [2] in order to guarantee that both discretizations converge to the right solution upon mesh refinement. Other techniques to enforce the constraints are related with the introduction of a penalty term which associates a high cost to the violation of the compatibility constraint. This is the case of penalty methods which have the advantage of not incorporating extra DOFs to the system of equations although the penalty term can influence the solution. Methods based on Augmented Lagrange multipliers seek for an optimal compromise between penalty and Lagrange multipliers allowing an exact enforcement in combination with a penalty-like regularization which improves the numerical treatment. In such methods the constraint violation is also penalized but, quite in contrast, the solution is not influenced by the penalty term. In fact, the convexity of the functional is increased to facilitate the search of its minimum. Explicit elimination of the constraints can be performed as well leading to a system of equations with no extra unknowns. However, these methods are not straightforward to implement for the case of non-matching meshes since they require to compute a null space of the compatibility matrices used in the equivalent dual formulation or constructing a projection operator which can be demanding in terms of storage. The reader is referred to the work of Rixen [45] for an overview of such techniques in the context of domain decomposition methods. Yet another method to enforce the constraints without the introduction of Lagrange multipliers was introduced by Nitsche [37] which can be regarded as an intermediate technique between the Lagrange multiplier and the penalty method. It essentially modifies the weak form by adding a term including a positive constant parameter that enforces the Dirichlet boundary condition. Such modification depends on the particular problem but it does not lead to an ill-conditioned system as the penalty method would do for large values of the penalty parameter. In fact the stabilization term exhibits less influence on the solution than standard penalty methods and, in practice, large values are not needed in order to ensure convergence and a proper enforcement of the constraints. A Nitsche method to handle the interface constraints derived from domain decomposition methods was introduced in [4]. In order to avoid integrating products of functions on unrelated meshes, the Lagrange multiplier method can be adopted to enforce the interface constraints and a Nitsche method can be employed to stabilize the system. This is accomplished by introducing an extra term in the variational principle that couples the multipliers with the stress fields at the interface [23]. Although the system of equations is augmented due to the introduction of the Lagrange unknowns, no constraints are needed for the discretization of the hybrid solution field since stabilization is accounted for by the extra “penalty” term. This technique has proven to be specially useful for the constraint enforcement in contact domain methods [21, 38] and will be utilized in the present contribution.

Domain decomposition frameworks typically found in literature that account for non-conforming interfaces are based on the introduction of Lagrange multipliers to weakly enforce the compatibility constraints. This is the case for the mortar approach [5] which is currently the most general and well established methodology. It essentially consists on a segment-to-segment discretization strategy where one of the domain surfaces at the interface is considered the ‘mortar’ (master) surface whilst the other is the ‘non-mortar’ (or slave). There are also variants of the approach where a third intermediate surface is considered with a reference displacement, however it obviously leads to an increase of the number of DOFs. Constraints are, therefore, weakly enforced by minimizing the gap with respect to the mortar surface. This methodology presents an obvious disadvantage when the selected mortar discretization is significantly coarser than the non-mortar one since a higher error can be obtained at the interface and might not satisfy the patch test. The dual domain decomposition method proposed by Herry et al. [24] presents a highly accurate technique to glue non-conforming interfaces by means of Lagrange Multipliers. It basically uses a third interface discretization with optimal location of the DOFs such that the kinematic continuity at the interface is exact. However, the technique is only valid for geometrically compatible non-conforming interfaces and, for this reason, arbitrary curved interfaces and interfaces which do not share the limit nodes can not be considered therein. The localized Lagrange multipliers (LLM) method proposed by Park et al. [39] consists in the introduction of a third interface surface with a specific discretization in order to collocate Lagrange multipliers to enforce the constraints at the non-matching meshes. Such discretization is performed in order to *a priori* satisfy the constant stress patch condition. The technique can be viewed as a general and optimal node-to-segment approach applied to the connection frame but still arbitrary highly irregular grids and geometrically incompatible interfaces are not addressed.

In contrast with the above mentioned techniques we propose a general and flexible methodology to account for the most complex interface situations, i.e. geometrically incompatible and arbitrary non-conforming. The main idea in the domain interface method (DIM) is to explicitly discretize the interface through a Delaunay triangulation. In all previously introduced techniques one slave node or segment belonging to a domain interface was somehow projected on the other domain interface or on an auxiliary one. Therefore, the interface constraints were formulated in a domain which is one dimension lower than the subdivided domains. In the DIM, the interface constraints are formulated on an intermediate interface of the same dimension as the adjacent decomposed domains (cf. Fig. 2). Consequently, the interface surface is continuous and uniquely defined upon the Delaunay discretization without any assumptions on the master/slave side. This results in full and non-overlapping connections leading to satisfactory results concerning the constant stress patch test. The geometrical details, weak form and FE implementation are given in Sect. 2 and a number of representative simulations are commented in Sect. 3 in order to highlight the advantages and applicability of the proposed approach against other established methodologies.

## Formulation of the DIM method

In this section the necessary geometrical aspects of the methodology are introduced and strong and weak forms of the problem are outlined. The solvability of the resulting system is discussed in terms of its stabilization and possible resolution choices including a parallel scheme. The main idea behind the DIM method concerns an explicit meshing of the interface between domains and is inspired in the methodology introduced in [21, 38] for contact mechanics. Rather than a particularization of the contact domain method for the case of tied contact the DIM equations stem from exporting the concept of the interface domain and the generality of the contact interface connections to the family of domain decomposition methods. In this manner, a new set of techniques within the domain decomposition methods is devised such as a non-intrusive methodology to handle rigid body modes (RBMs) without the need for extending the solution field to the RBM intensities as it is frequently done in established methodologies [11]. For the sake of completeness the methodology is introduced considering a finite strain case. Infinitesimal deformations are recovered by considering the necessary simplifications in the presented theory (i.e. small displacements compared to the domain dimensions and negligible gradients of such displacements). Compact notation will be utilized for tensor quantities throughout the document unless a different notation is specifically mentioned.

### Geometrical description of the DIM

**Fig. 3:**
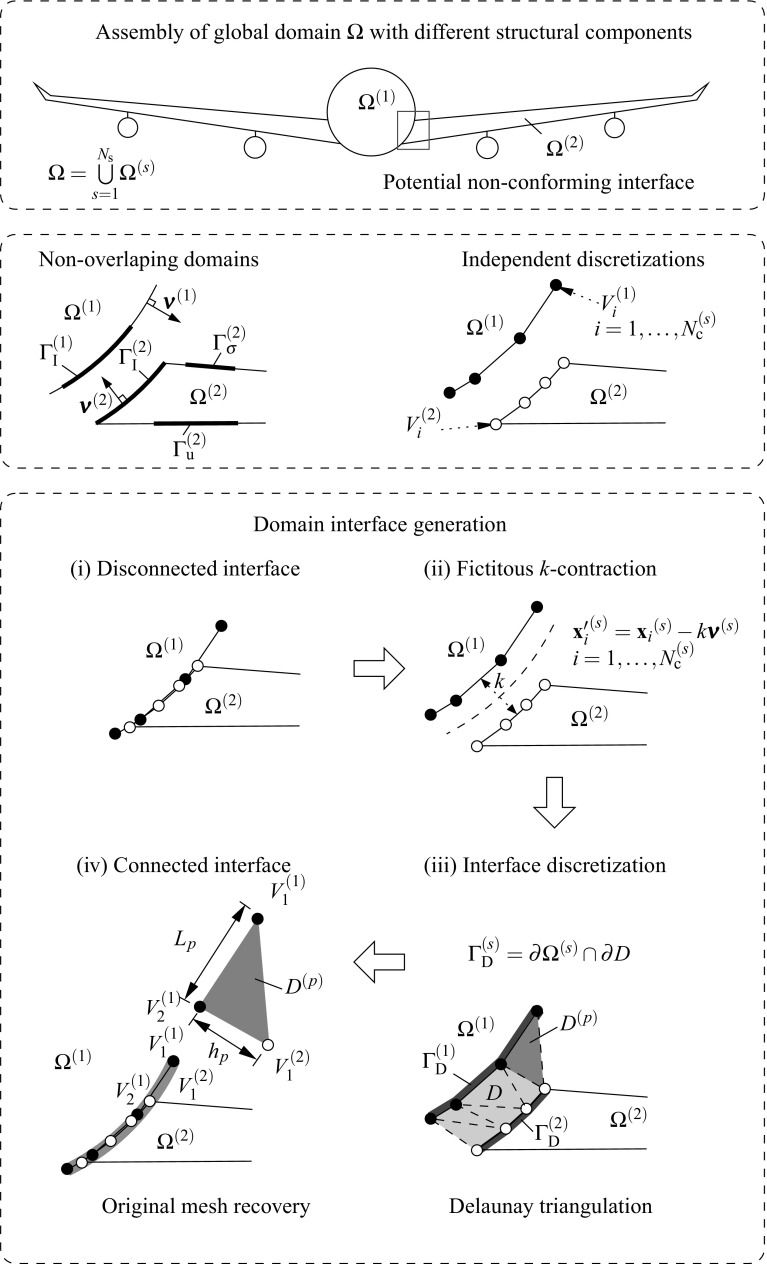
Generation of the domain interface: (*i*) domain discretizations, (*ii*) fictitious domain contractions, (*iii*) Delaunay triangulations and (*iv*) original mesh recovery

Consider the structure assembly depicted in Fig. 3 (top) where the domain $\Omega$ is composed by the union of $N_{\text {s}}$ non-overlapping domains $\Omega ^{(s)}$. At each domain $\Omega ^{(s)}$ one can identify the regions were Dirichlet $\Gamma _{\text {u}}^{(s)}$ and Neumann $\Gamma _{\sigma }^{(s)}$ boundary conditions are imposed. The interface $\Gamma _{\text {I}}^{(s)}=\partial \Omega ^{(s)} \cap \partial \Omega ^{(q)}$ with outward unit normal ${\pmb {\nu }}^{(s)}$ where $\partial \Omega$ stands for the domain boundaries of the adjacent domains *s* and *q*. Discretizations of the two bodies, e.g. using finite elements (FE), leads to a number of $N_{\lambda }^{(s)}$ vertices at the domain boundary $\partial \Omega ^{(s)}$ located at the vicinity of $\Gamma _{\text {I}}^{(s)}$ which need to be involved in the interface discretization.

The interface generation (cf. Fig. 3) starts with a fictitious contraction of the vertices $V_i$ in the direction $-{\pmb {\nu }}^{(s)}$ by a magnitude *k*. The result will be independent on the chosen magnitude *k* but in our analyses $k\approx h_{\text {e}}$, being $h_{\text {e}}$ an average of the equivalent FE size. The fictitious coordinates ${\mathbf {{x}}}^{\prime }_i$ are utilized to generate a Delaunay triangulation which defines the interface domain

1 $$\begin{aligned} D=\underset{p=1}{\overset{N_{\text {p}}}{\bigcup }} D^{(p)}, \end{aligned}$$

where $D^{(p)}$ represents each of the interface patches up to $N_{\text {p}}$. The interface surface $\Gamma _{\text {D}}^{(s)}=\partial \Omega ^{(s)} \cap \partial D$ and, for the case of a geometrically compatible interface, $\Gamma _{\text {I}}^{(s)}=\Gamma _{\text {D}}^{(s)}$.

#### *Remark 2.1*

Note that, upon discretization of the domain interface *D* using triangular linear elements $D^{(p)}$, the integrals over a geometrically incompatible interface when $h\rightarrow 0$ converge to a bounded value:

2 $$\begin{aligned} \displaystyle \sum _{p=1}^{N_{\text {p}}}\lim _{h_p\rightarrow 0} \int _{D^{(p)}} \dfrac{1}{h_p}(\bullet )\; \text {d} D= \dfrac{1}{2} \sum _{p=1}^{N_{\text {p}}}\int _{L^{(p)}}(\bullet )\; \text {d} L\,+\, \mathcal {O}(h), \end{aligned}$$

where $\mathcal {O}(h)$ corresponds to the error caused by approximating the interface geometry with piece-wise linear segments. Considering the case of geometrically compatible interfaces $\mathcal {O}(h)\rightarrow 0$ and

3 $$\begin{aligned} \displaystyle \sum _{p=1}^{N_{\text {p}}}\lim _{h_p\rightarrow 0} \int _{D^{(p)}} \dfrac{1}{h_p}(\bullet )\; \text {d} D\approx \dfrac{1}{2} \sum _{p=1}^{N_{\text {p}}}\int _{L^{(p)}}(\bullet )\; \text {d} L. \end{aligned}$$

Since the integration is performed over $D^{(p)}$ when $h\rightarrow 0$ it is pointed out that both adjacent domain interfaces $\Gamma _{\text {D}}^{(s)}$ and $\Gamma _{\text {D}}^{(q)}$ are automatically accounted for. This is in contrast with other methodologies, e.g. the mortar method [5], where only one of the adjacent interface discretizations is considered.

Consider the assembly of two non-overlapping domains $\Omega ^{(s)}$, $s=1,2$, (cf. Fig. 3) undergoing finite strains. The corresponding deformation maps are denoted by $\phi _t^{(s)}({\mathbf {{X}}})\equiv \phi ^{(s)}({\mathbf {{X}}},t)\; : \; \Omega _{0}^{(s)}\times [0,T]\rightarrow \Omega _{t}^{(s)}$, where material points ${\mathbf {{X}}}^{(s)}\in \Omega _{0}^{(s)}$ at the reference configuration are mapped onto the current configuration ${\mathbf {{x}}}^{(s)}=\phi _t^{(s)}({\mathbf {{X}}})\in \Omega _{t}^{(s)}$. Such a mapping can be expressed in terms of the total displacement field ${\mathbf {{U}}}_{t}^{(s)}({\mathbf {{X}}}^{(s)})$ which denotes the difference between reference and current coordinates satisfying

4 $$\begin{aligned} {\mathbf {{x}}}^{(s)}=\phi _t^{(s)}({\mathbf {{X}}})={\mathbf {{X}}}^{(s)}+{\mathbf {{U}}}_{t}^{(s)}({\mathbf {{X}}}^{(s)}). \end{aligned}$$

In our quasi-static analyses a pseudo-time domain $t\in [0,t]$ is considered with subdivisions in discrete intervals $[t_n,t_{n+1}]$ of incremental time length $\Delta t=t_{n+1}-t_n$. Configurations at the previous $t_n$ and current $t_{n+1}$ times are denoted as $\Omega _{n}^{(s)}=\phi _{t_n}^{(s)}(\Omega _{0}^{(s)})\equiv \phi _{n}^{(s)}(\Omega _{0}^{(s)})$ and $\Omega _{n+1}^{(s)}=\phi _{t_{n+1}}^{(s)}(\Omega _{0}^{(s)})\equiv \phi _{{n+1}}^{(s)}(\Omega _{0}^{(s)})$, respectively. Therefore, an expression of the incremental motion $\phi ^{(s)}$ can be found by substitution of the current and previous configurations in (4) as

**Fig. 4:**
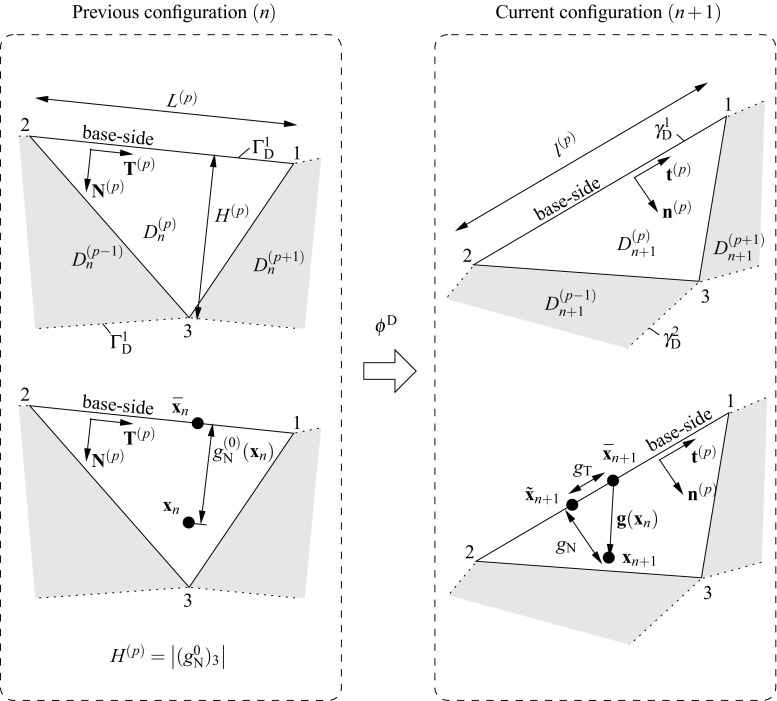
Geometrical definition of the gap ${\mathbf{g}}({\mathbf{x}}_n)$ according to the previous (*left*) and current (*right*) configurations

5 $$\begin{aligned} \left. \begin{aligned}&{\mathbf {{x}}}_{n+1}^{(s)}=\phi _{t_{n+1}}^{(s)}({\mathbf {{X}}}^{(s)})\\&{\mathbf {{x}}}_{n}^{(s)}=\phi _{t_{n}}^{(s)}({\mathbf {{X}}}^{(s)}) \end{aligned} \right\} \Rightarrow \begin{aligned}&{\mathbf {{x}}}_{n+1}^{(s)}=\phi _{n+1}^{(s)}\left( {(\phi _{n}^{(s)})^{-1}({\mathbf {{x}}}_{n}^{(s)})} \right) =\phi ^{(s)}({\mathbf {{x}}}_{n}^{(s)})\\&\forall {\mathbf {{x}}}_{n}^{(s)}\in \Omega _{n}^{(s)}. \end{aligned} \end{aligned}$$

With the incremental motion in hand, the incremental field

6 $$\begin{aligned} {\mathbf {{u}}}^{(s)}({\mathbf {{x}}}_n^{(s)})=\phi ^{(s)}({\mathbf {{x}}}_{n}^{(s)})-{\mathbf {{x}}}_{n}^{(s)}={\mathbf {{x}}}_{n+1}^{(s)}-{\mathbf {{x}}}_{n}^{(s)}, \quad \forall {\mathbf {{x}}}_{n}^{(s)}\in \Omega _n^{(s)}. \end{aligned}$$

The incremental motion of the interface domain

7 $$\begin{aligned} \phi ^{\text {D}}({\mathbf {{x}}}_n^{(s)})\equiv {\mathbf {{x}}}_{n+1}({\mathbf {{x}}}_{n})={\mathbf {{x}}}_{n}+{\mathbf {{u}}}^{\text {D}}({\mathbf {{x}}}_{n}), \quad \forall {\mathbf {{x}}}_{n}\in D_n^{(p)}, \end{aligned}$$

$D_n^{(p)}$ denoting the interface domain *D* at time $t_n$. Therefore, $D_{n+1}=\phi ^{\text {D}}(D_{n})$ and $\gamma _{\text {D}}=\phi ^{\text {D}}(\Gamma _{\text {D}})$ which represent the current and previous domain interface and interface surfaces, respectively (cf. Fig. 4). The incremental displacement field at the interface domain ${\mathbf {{u}}}^{\text {D}}$ is calculated by linearly interpolating the displacement increments $d_i^{\text {D}}$ of the interface element vertices as

8 $$\begin{aligned} {\mathbf {{u}}}^{\text {D}}({\mathbf {{x}}}_{n})\equiv {\mathbf {{u}}}^{(p)}({\mathbf {{x}}}_{n})=\sum _{i=1}^{3}\mathbb {N}_i({\mathbf {{x}}}_{n})d_i^{\text {D}}, \quad \forall {\mathbf {{x}}}_{n}\in D_n^{(p)}, \end{aligned}$$

$\mathbb {N}_i$ denoting linear shape functions for three-node triangular finite elements. The incremental gradient deformation tensor

9 $$\begin{aligned} {\mathbf {{f}}}^{\text {D}}=\bar{\pmb {\nabla }}\left( {\phi ^{\text {D}}({\mathbf {{x}}}_{n})} \right) =\dfrac{\partial {\mathbf {{x}}}_{n+1}}{\partial {\mathbf {{x}}}_{n}}={\mathbf{1}}+ \bar{\pmb {\nabla }}({\mathbf {{u}}}^{\text {D}}), \end{aligned}$$

where ${\mathbf {{1}}}$ represents de second order unity tensor and ${\bar{\pmb {\nabla }}}$ denotes the material gradient with respect to the reference previous configuration *n*.

#### *Remark 2.2*

It is important to note that ${\mathbf {{f}}}^{\text {D}}({\mathbf {{x}}}_{n})\equiv {\mathbf {{f}}}^{(p)}=constant, \quad \forall {\mathbf {{x}}}_{n}\in D^{(p)}$ due to the linear character of the incremental displacements defined in (8).

As depicted in Fig. 4, each interface patch $D^{(p)}$ contains a base-line defined on $\Gamma _{\text {D}}^{(p)}$ with unit normal vector ${\mathbf{N}}^{(p)}$ in the sense of the normal to the adjacent domain ${\pmb {\nu }}^{(s)}$. The tangential vector ${\mathbf{T}}^{(p)}=\hat{\mathbf{e}}\times {\mathbf{N}}^{(p)}$, where $\hat{\mathbf{e}}$ denotes the out-of-plane unit vector forming the ordered triplet of unit vectors $\left\{ {\mathbf{N}}^{(p)},{\mathbf{T}}^{(p)},\hat{\mathbf{e}}\right\}$. Considering the incremental motion $\phi ^{\text {D}}$, the current tangential and normal unit vectors read

10 $$\begin{aligned} \begin{aligned}&{\mathbf {{t}}}^{(p)}=\dfrac{\phi ^{\text {D}}({\mathbf {{T}}}^{(p)})}{\left| \left| \phi ^{\text {D}}({\mathbf {{T}}}^{(p)})\right| \right| }= \dfrac{{\mathbf{f}}^{(p)}\cdot {\mathbf {{T}}}^{(p)}}{\left| \left| {\mathbf{f}}^{(p)}\cdot {\mathbf {{T}}}^{(p)}\right| \right| }\\&{\mathbf {{n}}}^{(p)}={\mathbf {{t}}}^{(p)}\times \hat{\mathbf{e}}. \end{aligned} \end{aligned}$$

The definition of the normal and tangential vectors depends on the local base-line for every interface patch, therefore these vectors are constant within every patch (cf. 10) but discontinuous across the interface patches.

The initial normal gap $g_{\text {N}}^{0}$ (cf. Fig. 4) is defined at the previous configuration *n* for a given point ${\mathbf{x}}_n$ and its normal projection to the base-line $\bar{\mathbf{x}}_n$ as

11 $$\begin{aligned} g_{\text {N}}^{0}({\mathbf{x}}_n)=({\mathbf{x}}_n-\bar{\mathbf{x}}_n)\cdot {\mathbf{N}}^{(p)}. \end{aligned}$$

The final gap vector

12 $$\begin{aligned} {\mathbf {{g}}}({\mathbf{x}}_n)={\mathbf{x}}_{n+1}-\bar{\mathbf{x}}_{n+1}=\phi ^{\text {D}}({\mathbf{x}}_n)-\phi ^{\text {D}}(\bar{\mathbf{x}}_n), \end{aligned}$$

where ${\mathbf{x}}_{n+1}$ and $\bar{\mathbf{x}}_{n+1}$ stand for the convected points ${\mathbf{x}}_{n}$ and $\bar{\mathbf{x}}_{n}$, respectively. The final gap can be expressed as a sum of its normal and tangential projections onto the current base-side as

13 $$\begin{aligned} {\mathbf {{g}}}({\mathbf{x}}_n)&=g_{\text {N}}(\mathbf {{x}}_n){\mathbf {{n}}}^{(p)}\,+\,g_{\text {T}}(\mathbf {{x}}_n){\mathbf {{t}}}^{(p)}\nonumber \\&\Rightarrow \left\{ \begin{aligned}&g_{\text {N}}(\mathbf {{x}}_n)={\mathbf {{g}}}({\mathbf{x}}_n)\cdot {\mathbf {{n}}}^{(p)}\\&g_{\text {T}}(\mathbf {{x}}_n)={\mathbf {{g}}}({\mathbf{x}}_n)\cdot {\mathbf {{t}}}^{(p)}. \end{aligned} \right. \end{aligned}$$

Consequently, the normal gap $g_{\text {N}}(\mathbf {{x}}_n)$ can be seen as a projection in the direction of ${\mathbf {{n}}}^{(p)}$ which denotes penetration for negative values. Similarly, the tangential gap $g_{\text {T}}(\mathbf {{x}}_n)$ represents the slid distance in the sense of ${\mathbf {{t}}}^{(p)}$. It should be stressed that the gap definition utilized in this manuscript and already introduced in [21, 38] is not standard in the sense that it is defined as a patch-wise continuous function throughout the patch (*p*) and not only defined at the interface nodes as other methodologies would consider. For future use into the variational statement it is convenient to express the gap in terms of the displacement field and, to this end, a Taylor series expansion of $\phi ^{\text {D}}({\mathbf {{x}}}_n)$ is considered around $\bar{\mathbf {{x}}}_n$ up to second order terms, with ${\mathbf {{x}}}_n-\bar{\mathbf {{x}}}_n=g_{\text {N}}^{0}({\mathbf{x}}_n){\mathbf{N}}^{(p)}$. In this spirit

14 $$\begin{aligned} \begin{aligned} {\mathbf {{x}}_{n+1}}&=\phi ^{\text {D}}({\mathbf {{x}}}_n)=\phi ^{\text {D}}\left( {\bar{\mathbf {{x}}}_n+g_{\text {N}}^{0}({\mathbf{x}}_n)){\mathbf{N}}^{(p)}} \right) \\&=\phi ^{\text {D}}(\bar{\mathbf {{x}}}_n)+\bar{\pmb {\nabla }}\left( {\phi ^{\text {D}}(\bar{\mathbf {{x}}}_n)} \right) \cdot \left( {g_{\text {N}}^{0}({\mathbf{x}}_n)){\mathbf{N}}^{(p)}} \right) +\mathcal {O}({{\mathbf {{x}}}_n}^2)\\&=\bar{\mathbf {{x}}}_{n+1}+g_{\text {N}}^{0}({\mathbf{x}}_n)){\mathbf {{f}}}^{(p)}\cdot {\mathbf{N}}^{(p)}+\mathcal {O}({{\mathbf {{x}}}_n}^2). \end{aligned} \end{aligned}$$

Second order $\mathcal {O}({{\mathbf {{x}}}_n}^2)$ and higher order terms can be neglected due to the linear character of ${\mathbf {{f}}}^{(p)}$ and, therefore, the final expression for the gap vector as a function of the displacements reads

15 $$\begin{aligned} {\mathbf {{g}}}({\mathbf{x}}_n)= & {} {\mathbf{x}}_{n+1}-\bar{\mathbf{x}}_{n+1}=g_{\text {N}}^{0}({\mathbf{x}}_n)){\mathbf {{f}}}^{(p)}\cdot {\mathbf{N}}^{(p)}\nonumber \\= & {} g_{\text {N}}^{0}({\mathbf{x}}_n))\left( {{\mathbf{1}}+\bar{\pmb {\nabla }}({\mathbf {{u}}}^{(p)})} \right) \cdot {\mathbf{N}}^{(p)}. \end{aligned}$$

In the same spirit as in (13), the final gap ${\mathbf {{g}}}({\mathbf{x}}_n)=g_{\text {N}}(\mathbf {{x}}_n){\mathbf {{n}}}^{(p)}+g_{\text {T}}(\mathbf {{x}}_n){\mathbf {{t}}}^{(p)}$ with

16 $$\begin{aligned} \begin{aligned} g_{\text {N}}(\mathbf {{x}}_n)&={\mathbf {{n}}}^{(p)}\cdot {\mathbf {{g}}}({\mathbf{x}}_n)=g_{\text {N}}^{0}({\mathbf{x}}_n)){\mathbf{n}}^{(p)}\cdot {\mathbf {{f}}}^{(p)}\cdot {\mathbf{N}}^{(p)}\\&=g_{\text {N}}^{0}({\mathbf{x}}_n)){\mathbf{n}}^{(p)}\cdot \left( {{\mathbf{N}}^{(p)}+\bar{\pmb {\nabla }}({\mathbf {{u}}}^{(p)})\cdot {\mathbf{N}}^{(p)}} \right) ,\\ g_{\text {T}}(\mathbf {{x}}_n)&={\mathbf {{t}}}^{(p)}\cdot {\mathbf {{g}}}({\mathbf{x}}_n)=g_{\text {N}}^{0}({\mathbf{x}}_n)){\mathbf{t}}^{(p)}\cdot {\mathbf {{f}}}^{(p)}\cdot {\mathbf{N}}^{(p)}\\&=g_{\text {N}}^{0}({\mathbf{x}}_n)){\mathbf{t}}^{(p)}\cdot \left( {{\mathbf{N}}^{(p)}+\bar{\pmb {\nabla }}({\mathbf {{u}}}^{(p)})\cdot {\mathbf{N}}^{(p)}} \right) .\\ \end{aligned} \end{aligned}$$

#### *Remark 2.3*

The expressions of the gap match the ones obtained in node-to-segment techniques [51] when ${\mathbf {{x}}}_n$ is considered the slave node and $\bar{\mathbf {{x}}}_n$ is chosen as the master one. However, as already pointed out by Oliver et al. [38], the expressions in (16) can be regarded more general since they are defined continuously throughout the interface patch $D^{(p)}$ and not only for the vertices. Therefore the strategy is comparable to segment-to-segment techniques without the need for a definition of the master and slave surfaces.

In order to formulate the variational principle in Sect. 2.2 it is convenient to work with a dimensionless measure of the gap. In this view, the normal and tangential gap intensities are written relative to the absolute value of the original normal gap as

17 $$\begin{aligned} \bar{g}_{\text {N}}(\mathbf {{x}}_n) = \dfrac{{g}_{\text {N}}(\mathbf {{x}}_n)}{\left| {g}_{\text {N}}^{0}(\mathbf {{x}}_n)\right| } \quad \text {and} \quad \bar{g}_{\text {T}}(\mathbf {{x}}_n) = \dfrac{{g}_{\text {T}}(\mathbf {{x}}_n)}{\left| {g}_{\text {T}}^{0}(\mathbf {{x}}_n)\right| }. \end{aligned}$$

#### *Remark 2.4*

It is important to note that the gap intensity results singular when a perfect connection is fulfilled at the previous configuration, i.e. ${g}_{\text {T}}^{0}(\mathbf {{x}}_n)=0$. However, the integral terms added to the variational statement that account for the work at the interface converge to a bounded value despite the kernel being unbounded as shown in (2).

Inserting (17) in  (16) the final expression for the gap reads

18 $$\begin{aligned} \begin{aligned} {\bar{g}}_{\text {N}}(\mathbf {{x}}_n)&=sign\left( {g_{\text {N}}^{0}({\mathbf{x}}_n)} \right) {\mathbf{n}}^{(p)}\cdot \left( {{\mathbf{N}}^{(p)}+\bar{\pmb {\nabla }}({\mathbf {{u}}}^{(p)})\cdot {\mathbf{N}}^{(p)}} \right) ,\\ {\bar{g}}_{\text {T}}(\mathbf {{x}}_n)&=sign\left( {g_{\text {N}}^{0}({\mathbf{x}}_n)} \right) {\mathbf{t}}^{(p)}\cdot \left( {{\mathbf{N}}^{(p)}+\bar{\pmb {\nabla }}({\mathbf {{u}}}^{(p)})\cdot {\mathbf{N}}^{(p)}} \right) . \end{aligned} \end{aligned}$$

**Fig. 5:**
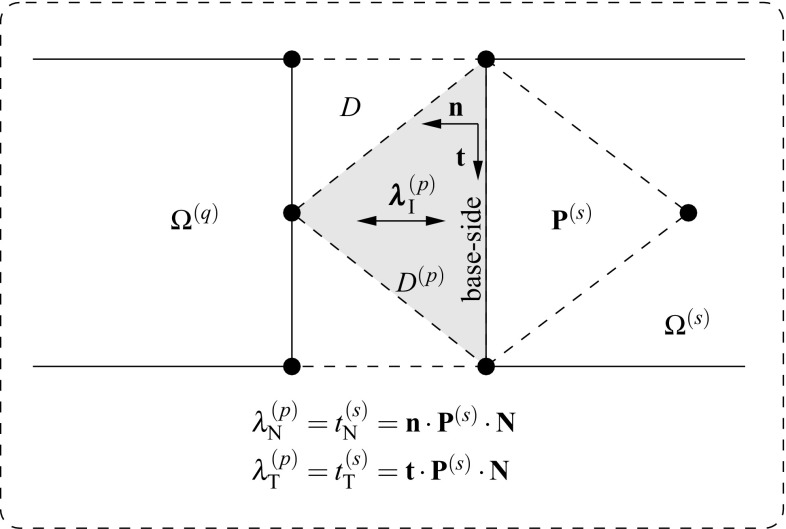
Lagrange multiplier identification at the interface

The interface traction vector acting at the surface $\Gamma _{\text {D}}^{(s)}$ can be expressed in terms of the first Piola–Kirchoff stress tensor ${\mathbf{P}}^{(s)}$ at time $n+1$ with respect to the configuration at time *n* and the normal vector ${\mathbf {{N}}}^{(p)}={\pmb {\nu }}_{n}^{(s)}$ as

19 $$\begin{aligned} {\mathbf{t}}_{\text {I}}({\mathbf {{x}}_n},{\mathbf {{N}}})^{(s)}={\mathbf{P}}^{(s)}\cdot {\mathbf {{N}}}^{(p)}, \quad \forall {\mathbf {{x}}}_n \in \Gamma _{\text {D}}^{(s)} \end{aligned}$$

with normal and tangential components w.r.t. the current normal and tangential vectors

20 $$\begin{aligned} \left. \begin{aligned} {t}_{\text {I,N}}(\mathbf {{x}}_n)&={\mathbf {{n}}}^{(p)}\cdot {\mathbf{P}}^{(s)}\cdot {\mathbf {{N}}}^{(p)}\\ {t}_{\text {I,T}}(\mathbf {{x}}_n)&={\mathbf {{t}}}^{(p)}\cdot {\mathbf{P}}^{(s)}\cdot {\mathbf {{N}}}^{(p)} \end{aligned} \right\} \quad \forall {\mathbf {{x}}}_n \in \Gamma _{\text {D}}^{(s)} \end{aligned}$$

and are defined at the surface $\gamma _{\text {D}}^{(s)}$ (cf. Fig. 4). Displacement compatibility between domains is enforced through the Lagrange multipliers defined constant at each patch $D^{(p)}$ as

21 $$\begin{aligned} \left. \begin{aligned} {\mathbf{\lambda }}_{\text {N}}(\mathbf {{x}}_n)&={t}_{\text {I,N}}(\mathbf {{x}}_n)\\ {\mathbf{\lambda }}_{\text {T}}(\mathbf {{x}}_n)&={t}_{\text {I,T}}(\mathbf {{x}}_n) \end{aligned} \right\} \quad \forall {\mathbf {{x}}}_n \in \Gamma _{\text {D}}^{(s)} \end{aligned}$$

which can be seen as the normal and tangential stresses that connect the adjacent domains (cf. Fig. 5).

#### *Remark 2.5*

For the case of tied contact within the present domain decompositon framework there is no need for a splitting between normal and tangential contributions. However, this splitting is introduced for the sake of completeness presenting a methodology that serves as a basis to tackle, if needed, more complex phenomena in which a different treatment can be considered for the tangential and normal interface components. Such scenarios may involve, for instance, sliding between domains. Additionally, the imposition of particular boundary conditions utilizing the Lagrange multiplier framework can be considered as well in which the tangential component is treated differently due to friction or sliding. Specific cases involving fluid structure interaction could in this manner be treated as well. The case of normal tying along an interface segment between fully tied limit corners has been studied already within a multiscale Domain Decomposition framework [30] to avoid undesirable stress concentrations at heterogeneous non-conforming interfaces. Such a splitting induces a non-linearity which would indeed have an impact within a small deformation setting but for the large strain formulation presented in this manuscript the price of the splitting is considered low compared to the benefits of increasing the applicability of the methodology.

### Strong and weak forms of the problem

The strong form of the equilibrium problem at domain $\Omega ^{(s)}$ can be written as:

22 $$\begin{aligned}&\text {FIND:}\left\{ \begin{aligned}&{\mathbf {{u}}}^{(s)}({\mathbf {{x}}}_{n}^{(s)}): \quad \Omega _{n}^{(s)}\rightarrow \mathbb {R}^{2}, \\&{\pmb {\lambda }}_{\text {I}}({\mathbf {{x}}}_{n})={\lambda }_{\text {N}}(\mathbf {{x}}_n){\mathbf {{n}}}^{(p)}+ {\lambda }_{\text {T}}(\mathbf {{x}}_n){\mathbf {{t}}}^{(p)} : \quad&D_{n}\rightarrow \mathbb {R}^{2}, \end{aligned} \right. \end{aligned}$$

23 $$\begin{aligned}&\quad \text {FULFILLING:}\nonumber \\&\text {Equilibrium equation:} \quad \bar{\pmb {\nabla }}\cdot {\mathbf{P}}^{(s)}={\mathbf {{0}}}, \quad \text {in } \Omega _{n}^{(s)} \end{aligned}$$

24 $$\begin{aligned}&\text {Constitutive model:} \quad {\mathbf{P}}^{(s)}={\pmb {\Sigma }}^{(s)}({\mathbf {{u}}}^{(s)}), \quad \text {in } \Omega _{n}^{(s)} \end{aligned}$$

25 $$\begin{aligned}&\text {Dirichlet's boundary conditions:}\quad {\mathbf{u}}^{(s)}={\hat{\mathbf{u}}}^{(s)}, \quad \text {in } \Gamma _{u}^{(s)} \end{aligned}$$

26 $$\begin{aligned}&\text {Neumann's boundary conditions:}\quad {\mathbf{t}}^{(s)}={\hat{\mathbf{t}}}^{(s)}, \quad \text {in } \Gamma _{\sigma }^{(s)} \end{aligned}$$

27 $$\begin{aligned}&\text {Lagrange multiplier identification:}\quad {\pmb {\lambda }}_{\text {I}}={\mathbf {{t}}}^{(s)},\quad \text {in }\Gamma _{\text {D}}^{(s)} \end{aligned}$$

28 $$\begin{aligned}&\text {Compatibility constraints:}\quad \left\{ \begin{aligned} \bar{g}_{\text {N}}(\mathbf {{u}}^{\text {D}})=0\\ \bar{g}_{\text {T}}(\mathbf {{u}}^{\text {D}})=0 \end{aligned} \right. ,\quad \text {in }D_{n}, \end{aligned}$$

${\bar{\pmb {\nabla }}}$ being the material gradient with respect to the previous configuration *n*, ${\mathbf{P}}^{(s)}$ the first Piola–Kirchoff corresponding to the previous configuration $\Omega _{n}^{(s)}$, ${\hat{\mathbf{t}}^{(s)}}$ and ${\hat{\mathbf{u}}^{(s)}}$ the prescribed tractions and displacements at $\Gamma _{\sigma }^{(s)}$ and $\Gamma _{u}^{(s)}$, respectively.

#### *Remark 2.6*

Note that the compatibility constraints are enforced to nullify the normal ${\bar{g}}_{\text {N}}(\mathbf {{u}}^{\text {D}})$ and tangential ${\bar{g}}_{\text {T}}(\mathbf {{u}}^{\text {D}})$ components of the effective gap satisfying, in this manner, displacement compatibility across domains as indicated in (28). The gaps are defined by means of the incremental displacements at the interface $\mathbf {{u}}^{\text {D}}$ which are calculated by interpolating the displacements at the vertices according to (8). In addition, the displacements at the vertices of the interface patch are calculated with the domain displacements $\mathbf {{u}}^{(s)}({\mathbf {{x}}}_n)$.

The weak form of the problem stated in (23–28) is expressed through the virtual work principle and variational statement of the constraint equations. To this end, the solution ${\pmb {\mathcal {V}}}$ and weighting spaces ${\pmb {\mathcal {V}}}_{0}$ for the displacement field as well as the Lagrange multiplier space ${\pmb {\mathcal {L}}}$ for the corresponding solution and weighting functions are defined as:

29 $$\begin{aligned}&{\pmb {\mathcal {V}}}:=\left\{ {\mathbf {{u}}}\Big /{\mathbf {{u}}}\Big |_{\Omega ^{(s)}} \in {\mathcal {H}}^{1}(\Omega ^{(s)}), \quad {\mathbf{u}}^{(s)}={\hat{\mathbf{u}}}^{(s)} \quad \text {in}\quad \Gamma _{u}^{(s)},\quad s:1\ldots N_{\text {s}}\right\} , \end{aligned}$$

30 $$\begin{aligned}&{\pmb {\mathcal {V}}}_{0}:=\left\{ \delta {\mathbf {{u}}}\Big / \delta {\mathbf {{u}}}\Big |_{\Omega ^{(s)}}\in {\mathcal {H}}^{1}(\Omega ^{(s)}), \quad \delta {\mathbf{u}}^{(s)}={\mathbf {{0}}} \quad \text {in}\quad \Gamma _{u}^{(s)}, \quad s:1\ldots N_{\text {s}}\right\} , \end{aligned}$$

31 $$\begin{aligned}&{\mathcal {L}}:=L^{2}(\text {D}), \end{aligned}$$

${\mathcal {H}}^{1}(\Omega ^{(s)})$ and ${{{L}}}^{2}(\text {D})$ being the Sovolev space of functions with square integrable derivatives and the Lebesgue space of square integrable functions, respectively. The variational statement reads:

32 $$\begin{aligned}&\text {FIND:}\left\{ \begin{aligned}&{\mathbf {{u}}}^{(s)}\in {\pmb {\mathcal {V}}}: \quad&\Omega _{n}^{(s)}\rightarrow \mathbb {R}^{2} \\&{\pmb {\lambda }}_{\text {I}}\in {\pmb {\mathcal {L}}}: \quad&D_{n}\rightarrow \mathbb {R}^{2} \end{aligned} \right. \end{aligned}$$

33 $$\begin{aligned}&\quad \text {FULFILLING:}\nonumber \\&\delta \Pi _{\text {mec}}({\mathbf {{u}}},{\pmb {\lambda }}_{\text {I}},\delta {\mathbf {{u}}}):=\delta \Pi _{\text {int,ext}}({\mathbf {{u}}},\delta {\mathbf {{u}}})+ \delta \Pi _{\text {I}}({\mathbf {{u}}},{\pmb {\lambda }}_{\text {I}},\delta {\mathbf {{u}}})=0, \nonumber \\&\quad \forall \delta {\mathbf {{u}}}^{(s)} \in {\pmb {\mathcal {V}}}_{0}. \end{aligned}$$

34 $$\begin{aligned}&\quad \text {AND}\nonumber \\&\delta \Pi _{\lambda _{\text {N}}}({\mathbf {{u}}}^{\text {D}},\delta {{\lambda }}_{\text {N}})=0 ,\quad \forall \delta {{\lambda }}_{\text {N}} \in {{\mathcal {L}}}, \end{aligned}$$

35 $$\begin{aligned}&\delta \Pi _{\lambda _{\text {T}}}({\mathbf {{u}}}^{\text {D}},\delta {{\lambda }}_{\text {T}})=0 ,\quad \forall \delta {{\lambda }}_{\text {T}} \in {{\mathcal {L}}}. \end{aligned}$$

The part of the mechanical work corresponding to internal and external forces

36 $$\begin{aligned} \delta \Pi _{\text {int,ext}}({\mathbf {{u}}},\delta {\mathbf {{u}}})=\delta \Pi _{\text {int}}({\mathbf {{u}}},\delta {\mathbf {{u}}})-\delta \Pi _{\text {ext}}(\delta {\mathbf {{u}}}) \end{aligned}$$

with

37 $$\begin{aligned} \delta \Pi _{\text {int}}({\mathbf {{u}}},\delta {\mathbf {{u}}})=\sum _{s=1}^{N_{s}}\left\{ \int _{\Omega _{n}^{(s)}}{\mathbf {{P}}}^{(s)}: \bar{\pmb {\nabla }}(\delta {\mathbf {{u}}}^{(s)})\;d\Omega \right\} \end{aligned}$$

and

38 $$\begin{aligned} \delta \Pi _{\text {ext}}(\delta {\mathbf {{u}}})=\sum _{s=1}^{N_{\text {s}}}\left\{ \int _{\Gamma _{\sigma }^{(s)}}\hat{\mathbf {{t}}}^{(s)}\cdot \delta {\mathbf {{u}}}^{(s)}\;d\Gamma \right\} . \end{aligned}$$

The work performed at the interface

39 $$\begin{aligned} \delta \Pi _{\text {I}}({\mathbf {{u}}},{\pmb {\lambda }}_{\text {I}})= & {} \int _{D_{n}}{\pmb {\lambda }}_{\text {I}}\cdot \delta {\bar{\mathbf {{g}}}}({\mathbf {{u}}}^{\text {D}})\;dD\nonumber \\= & {} \int _{D_{n}}{{\lambda }}_{\text {N}} \delta {\bar{{g}}}_{\text {N}}({\mathbf {{u}}}^{\text {D}})\;dD+\int _{D_{n}}{{\lambda }}_{\text {T}} \delta {\bar{{g}}}_{\text {T}}({\mathbf {{u}}}^{\text {D}})\;dD,\nonumber \\ \end{aligned}$$

where $\delta {\bar{\mathbf {{g}}}}$ denotes the gap intensity variations. It is worth noting that the resulting variational principles are considered on the interface volume (surface in 2D) $D_{n}$ and not at the interface surface (segments in 2D) as it is done in other established methodologies. Following the expressions of the gap intensity variations developed in [21, 38] the interface normal and tangential work contributions can be written in terms of the displacement variations as:

40 $$\begin{aligned} \int _{D_{n}}{{\lambda }}_{\text {N}} \delta {\bar{{g}}}_{\text {N}}({\mathbf {{u}}}^{\text {D}})\;dD&=\int _{D_{n}}{{\lambda }}_{\text {N}} {\bar{{g}}}_{\text {N}}{\mathbf {{n}}\cdot {\pmb {\nabla }}(\delta {\mathbf {{u}}}^{\text {D}})\cdot {\mathbf {{n}}}}\;dD, \end{aligned}$$

41 $$\begin{aligned} \int _{D_{n}}{{\lambda }}_{\text {T}} \delta {\bar{{g}}}_{\text {T}}({\mathbf {{u}}}^{\text {D}})\;dD&=\int _{D_{n}}{{\lambda }}_{\text {T}} {\bar{{g}}}_{\text {N}}{\mathbf {{n}}\cdot {\pmb {\nabla }}(\delta {\mathbf {{u}}}^{\text {D}})\cdot {\mathbf {{t}}}}\;dD\nonumber \\&\quad + \int _{D_{n}}{{\lambda }}_{\text {T}} {\bar{{g}}}_{\text {N}}{\mathbf {{t}}\cdot {\pmb {\nabla }}(\delta {\mathbf {{u}}}^{\text {D}})\cdot {\mathbf {{n}}}}\;dD\nonumber \\&\quad + \int _{D_{n}}{{\lambda }}_{\text {T}} {\bar{{g}}}_{\text {T}}{\mathbf {{t}}\cdot {\pmb {\nabla }}(\delta {\mathbf {{u}}}^{\text {D}})\cdot {\mathbf {{t}}}}\;dD, \end{aligned}$$

where ${{\pmb {\nabla }}}=\dfrac{\partial (\bullet )}{\partial {\mathbf {{x}}}_{n+1}}$ denotes the spatial gradient, i.e. taking derivatives with respect to the current configuration $n+1$.

Finally, the variational statements of the constraint equations

42 $$\begin{aligned} \delta \Pi _{\lambda _\text {N}}({\mathbf {{u}}}^{\text {D}},\delta {{\lambda }}_{\text {N}})&= \int _{D_{n}}\delta {{\lambda }}_{\text {N}}{\bar{g}}_{\text {N}}({\mathbf {{u}}}^{\text {D}})\;dD\nonumber \\&= \int _{D_{n}}\delta {{\lambda }}_{\text {N}}\, sign\left( {g_{\text {N}}^{0}} \right) {\mathbf{n}}\cdot \left( {{\mathbf{N}}+\bar{\pmb {\nabla }}({\mathbf {{u}}}^{\text {D}})\cdot {\mathbf{N}}} \right) \;dD, \end{aligned}$$

43 $$\begin{aligned} \delta \Pi _{\lambda _\text {T}}({\mathbf {{u}}}^{\text {D}},\delta {{\lambda }}_{\text {T}})&= \int _{D_{n}}\delta {{\lambda }}_{\text {T}}{\bar{g}}_{\text {T}}({\mathbf {{u}}}^{\text {D}})\;dD\nonumber \\&= \int _{D_{n}}\delta {{\lambda }}_{\text {T}}\, sign\left( {g_{\text {N}}^{0}} \right) {\mathbf{t}}\cdot \left( {{\mathbf{N}}+\bar{\pmb {\nabla }}({\mathbf {{u}}}^{\text {D}})\cdot {\mathbf{N}}} \right) \;dD, \end{aligned}$$

which force the interface work to nullify in an average sense along the domain interface $D_n$.

#### *Remark 2.7*

The equilibrium equation in (23) and imposed tractions at the boundary (26) correspond to the Euler–Lagrange equations and natural boundary conditions associated to the virtual work principle in (33). In the same spirit, the constraint equations in (28) correspond to the Euler–Lagrange equations associated to the constraint variational Eqs. (34, 35).

### Discretization using FE and lambda-solvability of the resulting system

Consider a Galerkin-based discretization in which the displacement solution field and its variations are interpolated using the shape functions $\mathbb {N}$ as:

44 $$\begin{aligned} {\mathbf {{u}}}^{(s)}({\mathbf {{x}}}_n)&=\sum _{a} {\mathbb {N}}_a({\mathbf {{x}}}_n){\mathbf {{d}}}_{a}^{(s)}\quad \forall {\mathbf {{x}}}_n \in \Omega _{n}^{(s)}, \end{aligned}$$

45 $$\begin{aligned} \delta {\mathbf {{u}}}^{(s)}({\mathbf {{x}}}_n)&=\sum _{a} {\mathbb {N}}_a({\mathbf {{x}}}_n)\delta {\mathbf {{d}}}_{a}^{(s)}\quad \forall {\mathbf {{x}}}_n \in \Omega _{n}^{(s)}, \end{aligned}$$

where the subscript *a* denotes the discrete nodes corresponding to the displacement interpolation. In a similar fashion, the displacements ${\mathbf {{u}}}^{\text {D}}$, Lagrange multipliers ${\pmb {\lambda }}_{\text {I}}$ and its corresponding variations at the interface patch *D* are discretized as:

46 $$\begin{aligned}&{\mathbf {{u}}}^{\text {D}}({\mathbf {{x}}}_n)=\sum _{a} {\mathbb {N}}_a({\mathbf {{x}}}_n){\mathbf {{d}}}_{a}^{\text {D}}\quad \forall {\mathbf {{x}}}_n \in D_n, \end{aligned}$$

47 $$\begin{aligned}&\delta {\mathbf {{u}}}^{\text {D}}({\mathbf {{x}}}_n)=\sum _{a} {\mathbb {N}}_a({\mathbf {{x}}}_n)\delta {\mathbf {{d}}}_{a}^{\text {D}}\quad \forall {\mathbf {{x}}}_n \in D_n, \end{aligned}$$

48 $$\begin{aligned}&{\pmb {\lambda }}_{\text {I}}({\mathbf {{x}}}_n)=\sum _{b} {\Psi }_{b}({\mathbf {{x}}}_n){\pmb {\Lambda }}_{b}\quad \forall {\mathbf {{x}}}_n \in D_n, \end{aligned}$$

49 $$\begin{aligned}&\delta {\pmb {\lambda }}_{\text {I}}({\mathbf {{x}}}_n)=\sum _{b} {\Psi }_{b}({\mathbf {{x}}}_n)\delta {\pmb {\Lambda }}_{b}\quad \forall {\mathbf {{x}}}_n \in D_n, \end{aligned}$$

where the subscript *b* stands for the the discrete nodes corresponding to the Lagrange multipliers interpolation using the shape functions ${\Psi }$ which read:

50 $$\begin{aligned} {\Psi }({\mathbf {{x}}}_n)=\left\{ \begin{aligned}&1 \quad \forall {\mathbf {{x}}}_n \in D_{n}^{(p)}\\&0 \quad \forall {\mathbf {{x}}}_n \not \in D_{n}^{(p)} \end{aligned} \right. . \end{aligned}$$

#### *Remark 2.8*

It should be noted that a piece-wise constant interpolation of the Lagrange multipliers might not lead to optimal spatial convergence rates. We have not observed any critical convergence behaviour in any of our simulations. However, it is observed in our analyses that the theoretical convergence rates might not be fully recovered due to use of piece-wise constant Lagrange multipliers. A more theoretical and practical study regarding convergence rates with respect to the choice of the Lagrange multiplier space is out of the scope of this work and could be considered as a future research topic for a more mathematically oriented contribution.

The FE approximation of the virtual work expression in (33) can be written using the above interpolations (44) to (49) as follows:

51 $$\begin{aligned}&\delta \Pi _{\text {mec}}({\mathbf {{u}}},{\pmb {\lambda }}_{\text {I}},\delta {\mathbf {{u}}})\approx \delta \Pi _{\text {mec}}^{\text {h}}({\mathbf {{d}}},{\pmb {\Lambda }},\delta {\mathbf {{d}}})= \delta \Pi _{\text {int,ext}}^{\text {h}}({\mathbf {{d}}},\delta {\mathbf {{d}}})\nonumber \\&\quad +\,\delta \Pi _{\text {I}}^{\text {h}}({\mathbf {{d}}},{\pmb {\Lambda }},\delta {\mathbf {{d}}}). \end{aligned}$$

Considering that (51) holds for any virtual displacement $\delta {\mathbf {{u}}}$, the residual forces of the variational principle

52 $$\begin{aligned} {\mathbf {{R}}}_{\text {mech}}({\mathbf {{d}}},{\pmb {\Lambda }}) = {\mathbf {{R}}}_{\text {int,ext}}({\mathbf {{d}}}) + {\mathbf {{R}}}_{\text {I}}({\mathbf {{d}}},{\pmb {\Lambda }}), \end{aligned}$$

with

53 $$\begin{aligned}&{\mathbf {{R}}}_{\text {int,ext}}({\mathbf {{d}}})=\sum _{s=1}^{N_{\text {s}}}\left( {{\mathbf {{F}}}_{\text {int}}^{(s)}({\mathbf {{d}}}^{(s)})-{\mathbf {{F}}}_{\text {ext}}^{(s)}} \right) \nonumber \\&\quad = \sum _{s=1}^{N_{\text {s}}}\left( \int _{\Omega ^{(s)}}\sum _{a}\left( {{\mathbf {{P}}}^{(s)}({\mathbf {{d}}}^{(s)})\cdot {\bar{\pmb {\nabla }}}({\mathbb {N}}_{a})} \right) \; d\Omega \right. \nonumber \\&\qquad \left. - \int _{\Omega ^{(s)}}\sum _{a}\left( {{{\mathbb {N}}_{a}}\hat{\mathbf {{t}}}_{a}^{(s)}} \right) \; d\Omega \right) , \end{aligned}$$

54 $$\begin{aligned}&\begin{aligned} {\mathbf {{R}}}_{\text {I}}({\mathbf {{d}}},{\pmb {\Lambda }})&= \int _{D_n} \sum _{b}\left( {{\Psi }_{b}{{\Lambda }}_{\text {N},b}} \right) {\bar{{g}}}_{\text {N}}{\mathbf {{n}} \left( {\sum _{a}{\pmb {\nabla }}({\mathbb {N}}_{a})} \right) \cdot {\mathbf {{n}}}}\; dD\\&\quad +\int _{D_n} \sum _{b}\left( {{\Psi }_{b}{{\Lambda }}_{\text {T},b}} \right) {\bar{{g}}}_{\text {N}}{\mathbf {{n}} \left( {\sum _{a}{\pmb {\nabla }}({\mathbb {N}}_{a})} \right) \cdot {\mathbf {{t}}}}\; dD\\&\quad +\int _{D_n} \sum _{b}\left( {{\Psi }_{b}{{\Lambda }}_{\text {T},b}} \right) {\bar{{g}}}_{\text {N}}{\mathbf {{t}} \left( {\sum _{a}{\pmb {\nabla }}({\mathbb {N}}_{a})} \right) \cdot {\mathbf {{n}}}}\; dD\\&\quad +\int _{D_n} \sum _{b}\left( {{\Psi }_{b}{{\Lambda }}_{\text {T},b}} \right) {\bar{{g}}}_{\text {T}}{\mathbf {{t}} \left( {\sum _{a}{\pmb {\nabla }}({\mathbb {N}}_{a})} \right) \cdot {\mathbf {{t}}}}\; dD, \end{aligned} \end{aligned}$$

where it is assumed that ${\mathbf {{\overline{g}}}}={\mathbf {\overline{{g}}}}({\mathbf {{d}}}^{\text {D}})$, ${\mathbf {{n}}}={\mathbf {{n}}}({\mathbf {{d}}}^{\text {D}})$ and ${\mathbf {{t}}}={\mathbf {{t}}}({\mathbf {{d}}}^{\text {D}})$.

Following an analogous procedure for the variational expression of the interface constraints (34, 35)

55 $$\begin{aligned}&\delta \Pi _{\lambda _\text {N}}({\mathbf {{u}}}^{\text {D}},\delta {{\lambda }}_{\text {N}})\approx \delta \Pi _{\lambda \text {N}}^{\text {h}}({\mathbf {{d}}}^{\text {D}},\delta {{\Lambda }_{\text {N}}}). \end{aligned}$$

56 $$\begin{aligned}&\delta \Pi _{\lambda _\text {T}}({\mathbf {{u}}}^{\text {D}},\delta {{\lambda }}_{\text {T}})\approx \delta \Pi _{\lambda \text {T}}^{\text {h}}({\mathbf {{d}}}^{\text {D}},\delta {{\Lambda }_{\text {T}}}). \end{aligned}$$

Considering that (55, 56) hold for any virtual Lagrange multiplier $\delta {{\lambda }}_{\text {N}}$ and $\delta {{\lambda }}_{\text {T}}$, the residual displacement gap of the constraint variational principle can be written in a matrix form as:

57 $$\begin{aligned} {\mathbf {{R}}}_{\lambda }({\mathbf {{d}}}^{\text {D}}) = \left[ \begin{array}{c} {R}_{\lambda _\text {N}}({\mathbf {{d}}}^{\text {D}})\\ {R}_{\lambda _\text {T}}({\mathbf {{d}}}^{\text {D}}) \end{array}\right] , \end{aligned}$$

with

58 $$\begin{aligned}&{R}_{\lambda _\text {N}}({\mathbf {{d}}}^{\text {D}})= \int _{D_n}sign(g_{\text {N}}^{0}){\mathbf {{n}}}\cdot \left( {{\mathbf {{N}}}+\left( {\sum _{a}\bar{\pmb {\nabla }}(\mathbb {N}_a{\mathbf {{d}}}_a^{\text {D}})\cdot {\mathbf {{N}}}} \right) } \right) \; dD, \end{aligned}$$

59 $$\begin{aligned}&{R}_{\lambda _\text {T}}({\mathbf {{d}}}^{\text {D}})= \int _{D_n}sign(g_{\text {N}}^{0}){\mathbf {{t}}}\cdot \left( {{\mathbf {{N}}}+\left( {\sum _{a}\bar{\pmb {\nabla }}(\mathbb {N}_a{\mathbf {{d}}}_a^{\text {D}})\cdot {\mathbf {{N}}}} \right) } \right) \; dD. \end{aligned}$$

The discrete problem to be solved can be specified in terms of the nodal quantities ${\mathbf {{d}}}$ and ${\pmb {\Lambda }}$ as:

60 $$\begin{aligned}&\text {FIND}\, {\mathbf {{d}}}\, \text {AND} \,{\pmb {\Lambda }}\, \text {FULFILLING} \nonumber \\&{\mathbf {{R}}}_{\text {mech}}({\mathbf {{d}}},{\pmb {\Lambda }}) ={\mathbf {{0}}}, \end{aligned}$$

61 $$\begin{aligned}&{\mathbf {{R}}}_{\lambda }({\mathbf {{d}}}^{\text {D}}) ={\mathbf {{0}}}. \end{aligned}$$

The set of Eqs. (60, 61) can be solved incrementally via a standard Newton-Raphson procedure on the linearized system

62 $$\begin{aligned} \left[ \begin{array}{l} {\mathbf {{R}}}_{\text {mech}}({\mathbf {{d}}},{\pmb {\Lambda }})\\ {\mathbf {{R}}}_{\lambda }({\mathbf {{d}}}) \end{array} \right] + \left[ \begin{array}{ll} \dfrac{\partial {\mathbf {{R}}}_{\text {mech}}({\mathbf {{d}}},{\pmb {\Lambda }})}{\partial {\mathbf {{d}}}} &{} \quad \dfrac{\partial {\mathbf {{R}}}_{\text {mech}}({\mathbf {{d}}},{\pmb {\Lambda }})}{\partial {\pmb {\Lambda }}}\\ \dfrac{\partial {\mathbf {{R}}}_{\lambda }({\mathbf {{d}}})}{\partial {\mathbf {{d}}}} &{} \quad {\mathbf {{0}}} \end{array} \right] \left[ \begin{array}{l} \Delta {\mathbf {{d}}}\\ \Delta {\pmb {\Lambda }} \end{array} \right] ={\mathbf {{0}}}, \end{aligned}$$

where $\Delta {\mathbf {{d}}}$ and $\Delta {\pmb {\Lambda }}$ denote the solution field increments to be solved at each iteration.

It is important to realize that zero entries appear at the tangent matrix due to the fact that the gap residual ${\mathbf {{R}}}_{\lambda }({\mathbf {{d}}})$ depends solely on the nodal displacements ${\mathbf {{d}}}$ and not on the Lagrange multipliers ${\pmb {\Lambda }}$. Consequently, the problem is prone to exhibit instabilities if the adopted solution field discretizations do not satisfy the Ladyzhenskaya–Babuška–Brezzi (LBB) condition [2]. In order to provide a dependency of the Lagrange multipliers ${\pmb {\Lambda }}$ for the gap residual ${\mathbf {{R}}}_{\lambda }({\mathbf {{d}}})$, a Nitsche method [37] is employed based on the work of Heintz and Hansbo [23] who introduced this methodology for the case of linear kinematics. The stabilization procedure utilized in this manuscript has been introduced by Oliver et al. [38] and Hartmann et al. [21] in the context of contact mechanics. It essentially consists in a modification of the constraint variational principles (34, 35) considering a weak format of the identities in (21) leading to

63 $$\begin{aligned} \delta \tilde{\Pi }_{\lambda _\text {N}}({\mathbf {{u}}}^{\text {D}},\delta {{\lambda }}_{\text {N}})&= \delta {\Pi }_{\lambda _\text {N}}({\mathbf {{u}}}^{\text {D}},\delta {{\lambda }}_{\text {N}})\nonumber \\&\quad + \int _{\partial D_{n}\cap \Gamma _{\text {D}}^{(s)}}\tau \delta {{\lambda }}_{\text {N}} \left( {{{t}}_{\text {N}}({\mathbf {{u}}}^{(s)})-{{\lambda }}_{\text {N}}} \right) \,d\Gamma = 0 ,\nonumber \\&\qquad \forall \delta {{\lambda }}_{\text {N}} \in {{\mathcal {L}}}_{\text {N}},\end{aligned}$$

64 $$\begin{aligned} \delta \tilde{\Pi }_{\lambda _\text {T}}({\mathbf {{u}}}^{\text {D}},\delta {{\lambda }}_{\text {T}})&= \delta {\Pi }_{\lambda _\text {T}}({\mathbf {{u}}}^{\text {D}},\delta {{\lambda }}_{\text {T}})\nonumber \\&\quad + \int _{\partial D_{n}\cap \Gamma _{\text {D}}^{(s)}}\tau \delta {{\lambda }}_{\text {T}} \left( {{{t}}_{\text {T}}({\mathbf {{u}}}^{(s)})-{{\lambda }}_{\text {T}}} \right) \;d\Gamma = 0 ,\nonumber \\&\qquad \forall \delta {{\lambda }}_{\text {T}} \in {{\mathcal {L}}}_{\text {T}}, \end{aligned}$$

with $\tau >0$ being an additional parameter that penalizes the violation of the identity expressed by the new term. In addition

65 $$\begin{aligned} \tau =\dfrac{\alpha _{\text {stab}}}{E_{\text {min}}}L, \end{aligned}$$

where $E_{\text {min}}$ is the minimal Young’s modulus of the adjacent domains, *L* stands for the base-side length of the interface domain element in the previous configuration (cf. Fig. 4) and $\alpha _{\text {stab}}$ corresponds to a dimensionless user defined parameter which is regarded independent of the mesh size [21]. Note that the units of the stabilization parameter $\tau$ are $[L]^3/[F]$ such that the additional variational term corresponds to an energetic contribution with units [*F*][*L*]. It should be noted that, since the penalized term is part of the Euler–Lagrange equations of the variational principle (27), it will tend to zero upon mesh refinement. For this reason the stabilization procedure described in (63, 64) is qualified as a consistent penalty method in which, unlike other non-consistent penalty methods, the parameter $\tau$ can be made significantly small without affecting the quality of the obtained results.

The new residual displacement gap of the modified variational constraints (63, 64)

66 $$\begin{aligned} {\tilde{\mathbf {{R}}}}_{\lambda }({\mathbf {{d}}}^{\text {D}},{\pmb {\Lambda }}) = \left[ \begin{array}{c} {\tilde{R}}_{\lambda _\text {N}}({\mathbf {{d}}}^{\text {D}},\Lambda _{\text {N}})\\ {\tilde{R}}_{\lambda _\text {T}}({\mathbf {{d}}}^{\text {D}},\Lambda _{\text {T}}) \end{array}\right] , \end{aligned}$$

with

67 $$\begin{aligned} {\tilde{R}}_{\lambda _\text {N}}({\mathbf {{d}}}^{\text {D}},\Lambda _{\text {N}})&= \int _{D_n}sign(g_{\text {N}}^{0}){\mathbf {{n}}}\cdot \left( {{\mathbf {{N}}}+\left( {\sum _{a}\bar{\pmb {\nabla }}(\mathbb {N}_a{\mathbf {{d}}}_a^{\text {D}})\cdot {\mathbf {{N}}}} \right) } \right) \; dD\nonumber \\&\quad +\int _{\Gamma _{n}}\tau \left( {{\mathbf {{n}}}\cdot {\mathbf {{P}}}^{(s)}\cdot {\mathbf {{N}}}-\sum _{b}\left( {{\Psi }_{b}{{\Lambda }}_{\text {N},b}} \right) } \right) \; d\Gamma \end{aligned}$$

68 $$\begin{aligned} {\tilde{R}}_{\lambda _\text {T}}({\mathbf {{d}}}^{\text {D}},\Lambda _{\text {T}})&= \int _{D_n}sign(g_{\text {N}}^{0}){\mathbf {{t}}}\cdot \left( {{\mathbf {{N}}}+\left( {\sum _{a}\bar{\pmb {\nabla }}(\mathbb {N}_a{\mathbf {{d}}}_a^{\text {D}})\cdot {\mathbf {{N}}}} \right) } \right) \; dD\nonumber \\&\quad +\int _{\Gamma _{n}}\tau \left( {{\mathbf {{t}}}\cdot {\mathbf {{P}}}^{(s)}\cdot {\mathbf {{N}}}-\sum _{b}\left( {{\Psi }_{b}{{\Lambda }}_{\text {T},b}} \right) } \right) \; d\Gamma . \end{aligned}$$

The first Piola–Kirchoff stress tensor ${\mathbf {{P}}}^{(s)}$ in (67, 68) belongs to domain $\Omega ^{(s)}$ and corresponds to the element adjacent to the base-line of the interface element $D^{(p)}$ (cf. Fig. 5). Taking into account the above expressions for the modified constraint residual, the system in (62) results in

69 $$\begin{aligned} \left[ \begin{array}{l} {\mathbf {{R}}}_{\text {mech}}({\mathbf {{d}}},{\pmb {\Lambda }})\\ {\tilde{\mathbf {{R}}}}_{\lambda }({\mathbf {{d}}},{\pmb {\Lambda }}) \end{array} \right] + \left[ \begin{array}{ll} \dfrac{\partial {\mathbf {{R}}}_{\text {mech}}({\mathbf {{d}}},{\pmb {\Lambda }})}{\partial {\mathbf {{d}}}} &{} \quad \dfrac{\partial {\mathbf {{R}}}_{\text {mech}}({\mathbf {{d}}},{\pmb {\Lambda }})}{\partial {\pmb {\Lambda }}}\\ \dfrac{\partial {\tilde{\mathbf {{R}}}}_{\lambda }({\mathbf {{d}}},{\pmb {\Lambda }})}{\partial {\mathbf {{d}}}} &{} \quad \dfrac{\partial {\tilde{\mathbf {{R}}}}_{\lambda }({\mathbf {{d}}},{\pmb {\Lambda }})}{\partial {\pmb {\Lambda }}} \end{array} \right] \left[ \begin{array}{l} \Delta {\mathbf {{d}}}\\ \Delta {\pmb {\Lambda }} \end{array} \right] ={\mathbf {{0}}}. \end{aligned}$$

#### *Remark 2.9*

The system in (69) is non-symmetric due to the fact that the stabilization term is only introduced in the constraint equations. The consistent symmetric version proposed by Heintz and Hansbo [23] could be utilized too and it would be recommended in those cases where the adopted FE model leads to a symmetric tangent stiffness matrix. In the context of a full parallel scheme, the symmetry of the system in (69) allows the use of efficient iterative solvers such as the preconditioned conjugate gradient [3].

### Parallel system resolution strategies

The dual assembly in (69) can be recast considering the discretized quantities for each domain $\Omega {(s)}$ in a matrix form as

70 $$\begin{aligned} \left[ \begin{array}{l@{\quad }l@{\quad }l@{\quad }l} {\mathbf {{K}}}_{\text {dd}}^{(1)} &{} \mathbf {{0}} &{} \mathbf {{0}} &{} {\mathbf {{K}}}_{\text {d}\Lambda }^{(1)} \\ \mathbf {{0}} &{} \ddots &{} \mathbf {{0}} &{} \vdots \\ \mathbf {{0}} &{} \mathbf {{0}} &{} {\mathbf {{K}}}_{\text {dd}}^{(N_{\text {s}})}&{} {\mathbf {{K}}}_{\text {d}\Lambda }^{(N_{\text {s}})}\\ {\mathbf {{K}}}_{\Lambda \text {d}}^{(1)} &{} \ldots &{} {\mathbf {{K}}}_{\Lambda \text {d}}^{(N_{\text {s}})}&{} {\mathbf {{K}}}_{\Lambda \Lambda } \\ \end{array}\right] \left[ \begin{array}{l} \Delta {\mathbf {{d}}}^{(1)} \\ \vdots \\ \Delta {\mathbf {{d}}}^{(N_{\text {s}})} \\ \Delta \pmb {\Lambda } \end{array}\right] = \left[ \begin{array}{l} {\mathbf {{r}}}_{\text {d}}^{(1)} \\ \vdots \\ {\mathbf {{r}}}_{\text {d}}^{(N_{\text {s}})}\\ {\mathbf {{r}}}_{\Lambda } \end{array}\right] , \end{aligned}$$

where

71 $$\begin{aligned}&\left[ \begin{array}{l@{\quad }l} {\mathbf {{K}}}_{\text {dd}}^{(s)} &{} {\mathbf {{K}}}_{\text {d}\Lambda }^{(s)} \\ {\mathbf {{K}}}_{\Lambda \text {d}}^{(s)} &{} {\mathbf {{K}}}_{\Lambda \Lambda } \\ \end{array}\right] = \left[ \begin{array}{l@{\quad }l} \dfrac{\partial \left( {{\mathbf {{R}}}_{\text {mech}}({\mathbf {{d}}},{\pmb {\Lambda }})} \right) ^{(s)}}{\partial {\mathbf {{d}}}^{(s)}} &{} \dfrac{\partial \left( {{\mathbf {{R}}}_{\text {mech}}({\mathbf {{d}}},{\pmb {\Lambda }})} \right) ^{(s)}}{\partial {\pmb {\Lambda }}^{(s)}}\\ \dfrac{\partial \left( {{\tilde{\mathbf {{R}}}}_{\lambda }({\mathbf {{d}}},{\pmb {\Lambda }})} \right) ^{(s)}}{\partial {\mathbf {{d}}}^{(s)}} &{} \dfrac{\partial {\tilde{\mathbf {{R}}}}_{\lambda }({\mathbf {{d}}},{\pmb {\Lambda }})}{\partial {\pmb {\Lambda }}} \end{array}\right] ,\end{aligned}$$

72 $$\begin{aligned}&\left[ \begin{array}{l} {\mathbf {{r}}}_{\text {d}}^{(s)} \\ {\mathbf {{r}}}_{\Lambda } \\ \end{array}\right] = \left[ \begin{array}{l} \left( {{\mathbf {{R}}}_{\text {mech}}({\mathbf {{d}}},{\pmb {\Lambda }})} \right) ^{(s)}\\ {\tilde{\mathbf {{R}}}}_{\lambda }({\mathbf {{d}}},{\pmb {\Lambda }}) \end{array}\right] \end{aligned}$$

and

73 $$\begin{aligned}&{\mathbf {{d}}}^{(s)}={\mathbf {{d}}}({\mathbf {{x}}}_n)|{\mathbf {{x}}_n}\in \Omega ^{(s)}, \end{aligned}$$

74 $$\begin{aligned}&{\pmb {\Lambda }}^{(s)}={{\pmb {\Lambda }}}({\mathbf {{x}}}_n)|{\mathbf {{x}}_n}\in \Gamma _{\text {I}}^{(s)}=\Gamma _{\text {I}}\cap \Omega ^{(s)}, \end{aligned}$$

75 $$\begin{aligned}&\left( {{\mathbf {{R}}}_{\text {mech}}({\mathbf {{d}}},{\pmb {\Lambda }})} \right) ^{(s)}={\mathbf {{R}}}_{\text {mech}}({\mathbf {{d}}},{\pmb {\Lambda }}),\quad \forall {\mathbf {{d}}}={\mathbf {{d}}}^{(s)}\quad \text {and} \quad \forall {\pmb {\Lambda }}={\pmb {\Lambda }}^{(s)}, \end{aligned}$$

76 $$\begin{aligned}&\left( {{\tilde{\mathbf {{R}}}}_{\lambda }({\mathbf {{d}}},{\pmb {\Lambda }})} \right) ^{(s)}={\tilde{\mathbf {{R}}}}_{\lambda }({\mathbf {{d}}},{\pmb {\Lambda }}),\quad \forall {\mathbf {{d}}}={\mathbf {{d}}}^{(s)}\quad \text {and} \quad \forall {\pmb {\Lambda }}={\pmb {\Lambda }}^{(s)}. \end{aligned}$$

The system in (70) is expected to be large and, therefore, suitable to be solved using a parallel scheme. Given a reasonable amount of memory, the sparse system in (70) can be tackled with a direct parallel solver using a moderate number of processors. These algorithms are referred to as multi-frontal or block-LU methods [46] and are based on independent simultaneous factorizations of the domain matrices. Although these techniques do not scale well in massively parallel computers, they provide the same robustness as traditional direct methods which make them attractive when dealing with general non-symmetric and ill-conditioned systems. Additionally, block-LU solvers account for automatic load-balancing and multi-threading which is specially effective when dealing with domains with a significant difference in terms of the number of DOF [9, 32].

Another option is to express the global system (70) in terms of the interface flexibility problem

77 $$\begin{aligned}&{\mathbf {{F}}}_{\text {I}}\Delta {\pmb {\Lambda }}={\Delta }{\mathbf {{g}}}_{\text {I}}, \end{aligned}$$

78 $$\begin{aligned}&{\mathbf {{F}}}_{\text {I}}={\mathbf {{K}}}_{\Lambda \Lambda }-{\sum _{s=1}^{N_{\text {s}}}}\left( {{\mathbf {{K}}}_{\Lambda \text {d}}^{(s)}\left( {{\mathbf {{K}}}_{\text {dd}}} \right) ^{-1}{\mathbf {{K}}}_{\text {d}\Lambda }^{(s)}} \right) , \end{aligned}$$

79 $$\begin{aligned}&{\Delta }{\mathbf {{g}}}_{\text {I}}={\mathbf {{r}}}_{\Lambda }-{\sum _{s=1}^{N_{\text {s}}}}\left( {{\mathbf {{K}}}_{\Lambda \text {d}}^{(s)}\left( {{\mathbf {{K}}}_{\text {dd}}} \right) ^{-1}{\mathbf {{r}}}_{\text {d}}^{(s)}} \right) , \end{aligned}$$

assuming that the matrices ${\mathbf {{K}}}_{\text {dd}}^{(s)}$ are not singular, i.e. they do not exhibit rigid body modes (RBMs) as observed in floating domains (cf. following Sect. 2.5 for a detailed explanation). The flexibility of the interface and the interface displacement gap increment are denoted by ${\mathbf {{F}}}_{\text {I}}$ and $\Delta {\mathbf {{g}}}_{\text {I}}$, respectively and can be interpreted as the condensation of the domain stiffness matrices and residual forces at the interface $\Gamma _{\text {I}}$. The domain displacement increments $\Delta {\mathbf {{d}}}^{(s)}$ can be independently calculated for each domain after the solution of the Lagrange multipliers $\Delta \pmb {\Lambda }$ as

80 $$\begin{aligned} \Delta {\mathbf {{d}}}^{(s)}=\left( {{\mathbf {{K}}}_{\text {dd}}^{(s)}} \right) ^{-1}\left( {{\mathbf {{r}}}_{\text {d}}^{(s)}-{\mathbf {{K}}}_{\text {d}\Lambda }^{(s)}\Delta \pmb {\Lambda }} \right) . \end{aligned}$$

In this second option, a blend of direct solvers are employed to independently compute the factorizations of the domain stiffness and an iterative solver is utilized for the solution of the interface problem in (77) as commonly done in most substructuring and domain decomposition techniques [10, 11]. Consequently, the domain stiffness factorizations, the resolution of the interface problem and the computation of the domain solution fields are inherently parallel tasks and, for this reason, the methodology scales well in massively parallel computers. Moreover, since the interface problem does not have to be explicitly assembled, the required memory profile for such a parallel resolution is significantly lower than for the parallel direct solution of the global system (70).

If the flexibility matrix ${\mathbf {{F}}}_{\text {I}}$ is symmetric, the interface problem in (77) can be solved by preconditioned Conjugate Gradient iterations, otherwise a Bi-Conjugate Gradient Stabilized (Bi-CGSTAB) or a Generalized Minimal Residual method (GMRES) can be employed [3]. In our case, the non-symmetry of the flexibility problem (77) can be caused by the stabilization procedure outlined in (63, 64), which only affects the constraint variational principle, or in those cases where the constitutive equations render a non-symmetric stiffness matrix. For the case of ill-conditioned systems, e.g. domains with high stiffness contrasts due to heterogeneous components or undergoing damage growth and coalescence, robust and efficient preconditioners are generally hard to find (cf. [29, 47, 49]).

The main goal of this contribution is not the parallel assessment of the proposed domain decomposition technique but rather the introduction of the novel concepts for handling non-conforming meshes and its performance in general assembly situations. However, it is highlighted that the algorithm is perfectly compatible with a full parallel scheme as the ones explained above. For clarity, in all the examples presented in Sect. 3 the flexibility problem in (77) was explicitly assembled and solved through standard direct solvers using an LU factorization.

### A non-intrusive strategy to handle rigid body modes in the DIM

If one or more subdomains $\Omega ^{(s)}$ exhibit rigid body modes (RBMs), the corresponding matrices ${\mathbf {{K}}}_{\text {dd}}^{(s)}$ are not invertible and the expressions of the interface problem in (77) to (79) need to be generalized in order to handle singular matrices ${\mathbf {{K}}}_{\text {dd}}^{(s)}$. In standard non-overlapping dual domain decomposition methods [11, 45], the expression for the nodal displacements at domain $\Omega ^{(s)}$ reads

81 $$\begin{aligned} \Delta {\mathbf {{d}}}^{(s)}=\left( {{\mathbf {{K}}}_{\text {dd}}^{(s)}} \right) ^{+}\left( {{\mathbf {{r}}}_{\text {d}}^{(s)}-{\mathbf {{K}}}_{\text {d}\Lambda }^{(s)}\Delta \Lambda } \right) -{\mathbf{R}}^{(s)}{\pmb {\alpha }}^{(s)}, \end{aligned}$$

${{\mathbf {{K}}}_{\text {dd}}^{(s)}}^{+}$, ${\mathbf{R}}^{(s)}$ and ${\pmb {\alpha }}^{(s)}$ being a generalized inverse, rigid body modes (RBMs) and their amplitudes, respectively. The RBMs ${\mathbf{R}}^{(s)}$ span the null space of ${\mathbf {{K}}}_{\text {dd}}^{(s)}$ and represent all displacement configurations that do not contribute to the deformation energy, thus satisfying ${\mathbf {{K}}}_{\text {dd}}^{(s)}\Delta {\mathbf {{d}}}_{\text {rbm}}={\mathbf{0}}$ at the corresponding domain $\Omega ^{(s)}$. Note that the generalized inverse ${{\mathbf {{K}}}_{\text {dd}}^{(s)}}^{+}$ coincides with the standard inverse ${{\mathbf {{K}}}_{\text {dd}}^{(s)}}^{-1}$ if ${\mathbf {{K}}}_{\text {dd}}^{(s)}$ is non-singular and fulfills

82 $$\begin{aligned}&{\mathbf {{K}}}_{\text {dd}}^{(s)} {{\mathbf {{K}}}_{\text {dd}}^{(s)}}^{+} {\mathbf {{K}}}_{\text {dd}}^{(s)}={\mathbf {{K}}}_{\text {dd}}^{(s)}, \end{aligned}$$

83 $$\begin{aligned}&{{\mathbf {{K}}}_{\text {dd}}^{(s)}}^{+} {\mathbf {{K}}}_{\text {dd}}^{(s)} {{\mathbf {{K}}}_{\text {dd}}^{(s)}}^{+}={{\mathbf {{K}}}_{\text {dd}}^{(s)}}^{+}. \end{aligned}$$

The computation of the generalized inverse can be performed by zeroing the rows and columns corresponding to a zero pivot as in the temporary links method [16]. The rigid body modes ${\mathbf{R}}^{(s)}$ can be computed as a by-product of the factorization of ${\mathbf {{K}}}_{\text {dd}}^{(s)}$ or by geometrical inspection considering the given Dirichlet boundary conditions.

The mechanical work developed by the domain boundary forces ${\mathbf {{r}}}_{\text {d}}^{(s)}-{\mathbf {{K}}}_{\text {d}\Lambda }^{(s)}\Delta \varvec{\Lambda }$ must vanish in the directions of the rigid body modes and, therefore the following orthogonality condition must be satisfied

84 $$\begin{aligned} {{\mathbf{R}}^{(s)}}^\text {T}\left( {{\mathbf {{r}}}_{\text {d}}^{(s)}-{\mathbf {{K}}}_{\text {d}\Lambda }^{(s)}\Delta \pmb {\Lambda }} \right) ={\mathbf{0}}. \end{aligned}$$

Substituting (81) in the stabilized constraint conditions assembled in (70)

85 $$\begin{aligned} \sum _{s=1}^{N_{\text {s}}}{\mathbf {{K}}}_{\Lambda \text {d}}^{(s)}{\Delta {\mathbf {{d}}}^{(s)}}+{\mathbf {{K}}}_{\Lambda \Lambda }\Delta \pmb {\Lambda }={\mathbf {{r}}}_{\Lambda } \end{aligned}$$

and taking into account the orthogonality condition (84), the following general expression for the interface problem is obtained:

86 $$\begin{aligned} \left[ \begin{array}{l@{\quad }l} {\mathbf{F}}_{\text {I}}&{}{\mathbf{G}}_{\text {I}}\\ {{\mathbf{G}}_{\text {I}}}^\text {T}&{}{\mathbf{0}} \end{array}\right] \left[ \begin{array}{l} \Delta \pmb {\Lambda }\\ \Delta \pmb {\alpha } \end{array}\right] = \left[ \begin{array}{l} \Delta {\mathbf{g}}_{\text {I}}\\ \Delta {\mathbf{e}} \end{array}\right] , \end{aligned}$$

where

87 $$\begin{aligned}&{\mathbf{F}}_{\text {I}}=\sum _{s=1}^{{N}_{\text {s}}} {\mathbf{K}}_{\Lambda \text {d}}^{(s)}{\mathbf{K}}_{\text {dd}}^{{(s)}^{+}}{\mathbf{K}}_{\text {d}\Lambda }^{{(s)}^\text {T}}, \end{aligned}$$

88 $$\begin{aligned}&\Delta {\mathbf{g}}_{\text {I}}=\sum _{s=1}^{{N}_{\text {s}}} {\mathbf{K}}_{\Lambda \text {d}}^{(s)}{\mathbf{K}}_{\text {dd}}^{{(s)}^{+}}{\mathbf{r}}_{\text {d}}^{(s)}+ {\mathbf {{K}}}_{\Lambda \Lambda }\Delta \pmb {\Lambda }-{\mathbf {{r}}}_{\Lambda }, \end{aligned}$$

89 $$\begin{aligned}&{\mathbf{G}}_{\text {I}}=\left[ \begin{array}{ccc} {\mathbf{K}}_{\Lambda \text {d}}^{(1)}{\mathbf{R}}^{(1)}&\ldots&{\mathbf{K}}_{\Lambda \text {d}}^{({N}_{\text {s}})}{\mathbf{R}}^{({N}_{\text {s}})} \end{array}\right] , \end{aligned}$$

90 $$\begin{aligned}&\pmb {\alpha }=\left[ \begin{array}{ccc} \pmb {\alpha }^{{(1)}^\text {T}}&\ldots&\pmb {\alpha }^{{({N}_{\text {s}})}^\text {T}} \end{array}\right] ^{\text {T}}, \quad \text {and} \end{aligned}$$

91 $$\begin{aligned}&\Delta {\mathbf{e}}=\left[ \begin{array}{ccc} {\mathbf{r}}_{\text {d}}^{{(1)}^\text {T}}{\mathbf{R}}^{(1)}&\ldots&{\mathbf{r}}_{\text {d}}{\mathbf{f}}^{{({N}_{\text {s}})}^\text {T}}{\mathbf{R}}^{({N}_{\text {s}})} \end{array}\right] ^{\text {T}}. \end{aligned}$$

In the augmented interface system (86) the operator ${\mathbf{G}}_{\text {I}}$ is built considering the rigid body modes of each domain restricted onto the interface, i.e. considering only those configurations of the rigid body modes with components at the interface and neglecting all inner degrees of freedom. The increments $\Delta {\mathbf{e}}$ correspond to the residual at the interface which is out of balance with respect to the rigid body modes.

The local problems in (81) are solved using direct solvers while an iterative solver is employed for the augmented interface problem (86) which is transformed into a semi-definite system of equations on $\Delta {\pmb {\Lambda }}$, i.e. eliminating the RBMs from the system, by imposing ${\mathbf {{G}}}_{\text {I}}^{\text {T}}\Delta {\pmb {\Lambda }}={\mathbf {{e}}}$ through a projection operator (cf. [12, 45] for a more detailed explanation).

Although the methodology presented in (81) to (91) is general for the parallel processing of any dual system (70), its implementation in commercial FE packages is regarded highly intrusive. For this reason, a new methodology is proposed in the context of the DIM method which is capable of handling rigid body modes without the need for augmenting the flexibility system (77). The method essentially adds a new term to the virtual mechanical work expression (33) which penalizes a function of the type $\frac{1}{2}\left| \left| \bar{\mathbf {{g}}}\right| \right| ^2$. The new term is nullified in the solution since it is related with the Euler–Lagrange equations of the constraint variational principle and, therefore motivates the character of interior penalty method of the proposed procedure in the sense that mesh refinement will force the penalized term to tend to zero. The new virtual work expression reads:

92 $$\begin{aligned} \delta \tilde{\Pi }_{\text {mec}}({\mathbf {{u}}},{\pmb {\lambda }}_{\text {I}},\delta {\mathbf {{u}}})&:=\delta {\Pi }_{\text {mec}}({\mathbf {{u}}},{\pmb {\lambda }}_{\text {I}},\delta {\mathbf {{u}}})\nonumber \\&\quad \quad + \delta {\Pi }_{\text {RBM}}({\mathbf {{u}}}^{\text {D}},\delta {\mathbf {{u}}}^{\text {D}})=0, \end{aligned}$$

93 $$\begin{aligned} \delta {\Pi }_{\text {RBM}}({\mathbf {{u}}}^{\text {D}},\delta {\mathbf {{u}}}^{\text {D}})&=\sum _{r=1}^{N_{\text {r}}}c\int _{\Gamma _{\text {D}}^{(r)}}\delta {\bar{\mathbf {{g}}}}\cdot {\bar{\mathbf {{g}}}}\;d\Gamma \nonumber \\&= \sum _{r=1}^{N_{\text {r}}}c\int _{\Gamma _{\text {D}}^{(r)}}\delta {\bar{{g}}}_{\text {N}}{\bar{{g}}}_{\text {N}}\;d\Gamma \nonumber \\&\quad + \sum _{r=1}^{N_{\text {r}}}c\int _{\Gamma _{\text {D}}^{(r)}}\delta {\bar{{g}}}_{\text {T}}{\bar{{g}}}_{\text {T}}\;d\Gamma , \end{aligned}$$

**Fig. 6:**
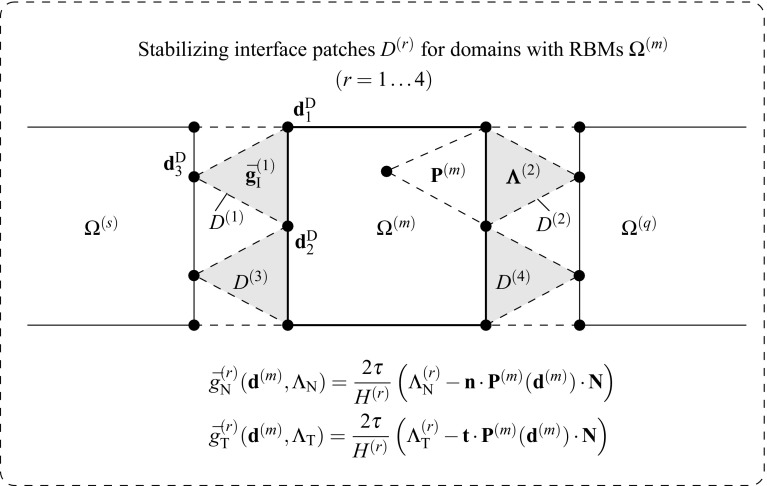
Handling domains $\Omega ^{(m)}$ with RBMs. Stabilizing interface patches $D^{(r)},\; r=1\ldots 4$ correspond to the *shadowed elements*

$\Gamma _{\text {D}}^{(r)}$ being the interface segments such that $\Gamma _{\text {D}}^{(r)}=\Omega ^{(s)}\cap D^{(r)}$, $N_{\text {r}}$ representing the number of patches utilized to handle the RBMs (cf. Fig. 6) and *c* denoting the penalty coefficient utilized to enforce the new condition. The additional term (93) can be expressed in terms of the virtual displacements as

94 $$\begin{aligned}&\sum _{r=1}^{N_{\text {r}}}c\int _{\Gamma _{\text {D}}^{(r)}}\delta {\bar{{g}}}_{\text {N}}{\bar{{g}}}_{\text {N}}\;d\Gamma = \sum _{r=1}^{N_{\text {r}}}c\int _{\Gamma _{\text {D}}^{(r)}} \left( {{\bar{{g}}}_{\text {N}}{\mathbf {{n}}\cdot {\pmb {\nabla }}(\delta {\mathbf {{u}}}^{\text {D}})\cdot {\mathbf {{n}}}}} \right) {\bar{{g}}}_{\text {N}}\;d\Gamma , \end{aligned}$$

95 $$\begin{aligned}&\sum _{r=1}^{N_{\text {r}}}c\int _{\Gamma _{\text {D}}^{(r)}}\delta {\bar{{g}}}_{\text {T}}{\bar{{g}}}_{\text {T}}\;d\Gamma = \sum _{r=1}^{N_{\text {r}}}c\int _{\Gamma _{\text {D}}^{(r)}} \left( {\bar{{g}}}_{\text {N}}{\mathbf {{n}}\cdot {\pmb {\nabla }}(\delta {\mathbf {{u}}}^{\text {D}})\cdot {\mathbf {{t}}}}\right. \nonumber \\ {}&\quad \left. + {\bar{{g}}}_{\text {N}}{\mathbf {{t}}\cdot {\pmb {\nabla }}(\delta {\mathbf {{u}}}^{\text {D}})\cdot {\mathbf {{n}}}} +\, {\bar{{g}}}_{\text {T}}{\mathbf {{t}}\cdot {\pmb {\nabla }}(\delta {\mathbf {{u}}}^{\text {D}})\cdot {\mathbf {{t}}}}\right) {\bar{{g}}}_{\text {T}}\;d\Gamma . \end{aligned}$$

Considering that Eqs. (94, 95) hold for any virtual displacement $\delta {\mathbf {{u}}}^{\text {D}}$, the corresponding residual

96 $$\begin{aligned}&{\mathbf {{R}}}_{\text {RBM}}({\mathbf {{d}}}^{\text {D}})={\mathbf {{R}}}_{\text {RBM},N}({\mathbf {{d}}}^{\text {D}})+{\mathbf {{R}}}_{\text {RBM},T}({\mathbf {{d}}}^{\text {D}}), \end{aligned}$$

97 $$\begin{aligned}&{\mathbf {{R}}}_{\text {RBM},N}({\mathbf {{d}}}^{\text {D}})=\sum _{r=1}^{N_{\text {r}}}c\int _{\Gamma _{\text {D}}^{(r)}}\left( {{\bar{{g}}}_{\text {N}}{\mathbf {{n}} \left( {\sum _{a}{\pmb {\nabla }}({\mathbb {N}}_{a})} \right) \cdot {\mathbf {{n}}}}} \right) {\bar{{g}}}_{\text {N}}\;d\Gamma , \end{aligned}$$

98 $$\begin{aligned} {\mathbf {{R}}}_{\text {RBM},T}({\mathbf {{d}}}^{\text {D}})&=\sum _{r=1}^{N_{\text {r}}}c\int _{\Gamma _{\text {D}}^{(r)}} \left( {\bar{{g}}}_{\text {N}}{\mathbf {{n}} \left( {\sum _{a}{\pmb {\nabla }}({\mathbb {N}}_{a})} \right) \cdot {\mathbf {{t}}}}\right. \nonumber \\&\quad \left. +\, {\bar{{g}}}_{\text {N}}{\mathbf {{t}} \left( {\sum _{a}{\pmb {\nabla }}({\mathbb {N}}_{a})} \right) \cdot {\mathbf {{n}}}}\;+\; {\bar{{g}}}_{\text {T}}{\mathbf {{t}} \left( {\sum _{a}{\pmb {\nabla }}({\mathbb {N}}_{a})} \right) \cdot {\mathbf {{t}}}}\right) {\bar{{g}}}_{\text {T}}\;d\Gamma . \end{aligned}$$

Note that both ${\bar{{g}}}_{\text {N}}$ and ${\bar{{g}}}_{\text {T}}$ are a function of ${\mathbf {{d}}}^{\text {D}}$ and, therefore

99 $$\begin{aligned} \dfrac{\partial {\mathbf {{R}}}_{\text {RBM}}({\mathbf {{d}}}^{\text {D}})}{\partial {\mathbf {{d}}}}&=f({\mathbf {{d}}}^{\text {D}}), \end{aligned}$$

100 $$\begin{aligned} \dfrac{\partial {\mathbf {{R}}}_{\text {RBM}}({\mathbf {{d}}}^{\text {D}})}{\partial {\pmb {\Lambda }}}&={\mathbf {{0}}}. \end{aligned}$$

Consequently, there will be contributions to the matrix ${\mathbf {{K}}}_{\text {dd}}^{(s)}$, being $\Omega ^{(s)}$ the domain sharing the segment $\Gamma _{\text {D}}^{(r)}$ with the patch $D^{(r)}$, and also to the adjacent domain $\Omega ^{(q)}$ (cf. Fig. 6) which will brake the band structure detailed in (70). In order to avoid the coupling of displacement quantities from adjacent domains it is proposed to express the gap as a function of the interface Lagrange multipliers and surface tractions on the same domain and substitute the expression in the residuals (97, 98).

To this end, we consider the stabilization expressions (63, 64). The use of linear triangular interface patches leads to patch-wise constant normal and tangential vectors ${\mathbf {{n}}}^{(r)},{\mathbf {{t}}}^{(r)}$ and gap intensities ${\bar{g}}_{\text {N}},{\bar{g}}_{\text {T}}$. Therefore the discretized residual Eqs. (67, 68) can be re-written as

101 $$\begin{aligned}&\int _{D^{(r)}}{\bar{g}}_{\text {N}}\; dD +\int _{\Gamma _{\text {D}}^{(r)}}\tau \left( {{t}_{\text {N}}-{\Lambda }_{\text {N}}^{(r)}} \right) \; d\Gamma =0, \nonumber \\&\qquad p\in \{1,\ldots ,N_{\text {p}}\}, \end{aligned}$$

102 $$\begin{aligned}&\int _{D^{(r)}}{\bar{g}}_{\text {T}}\; dD +\int _{\Gamma _{\text {D}}^{(r)}}\tau \left( {{t}_{\text {T}}-{\Lambda }_{\text {T}}^{(r)}} \right) \; d\Gamma =0, \nonumber \\&\qquad p\in \{1,\ldots ,N_{\text {p}}\}, \end{aligned}$$

where the base-side of the patch is denoted by $\Gamma _{\text {D}}^{(r)}$ (cf. Fig. 4). The volume and surface integrals in (101, 102) can be calculated as

103 $$\begin{aligned}&\int _{D^{(r)}}\; dD= \dfrac{1}{2}L^{(r)}H^{(r)}, \end{aligned}$$

104 $$\begin{aligned}&\int _{\Gamma _{\text {D}}^{(r)}}\; d\Gamma =L^{(r)}, \end{aligned}$$

$L^{(r)}$ being the length of the base-side and $H^{(r)}=\left| g_{\text {N}}^0(x_3)\right|$ the absolute value of the initial normal gap corresponding to the interface element vertex 3 (cf. Fig. 4) which is the height of the interface patch $D_n^{(r)}$ in the previous configuration. Considering the integrals (103, 104) and taking into account that $t_{\text {N}}$ and $t_{\text {T}}$ from the element adjacent to $D^{(r)}$ are constant, the expressions in (101, 102) yield

105 $$\begin{aligned}&\dfrac{1}{2}H^{(r)}{\bar{g}}_{\text {N}}+\tau \left( {{t}_{\text {N}}-{\Lambda }_{\text {N}}^{(r)}} \right) =0, \quad p\in \{1,\ldots ,N_{\text {p}}\}, \end{aligned}$$

106 $$\begin{aligned}&\dfrac{1}{2}H^{(r)}{\bar{g}}_{\text {T}}+\tau \left( {{t}_{\text {T}}-{\Lambda }_{\text {T}}^{(r)}} \right) =0. \quad p\in \{1,\ldots ,N_{\text {p}}\}, \end{aligned}$$

The effective gap components can be now expressed in terms of the Lagrange multiplier and traction vector components as

107 $$\begin{aligned}&{\bar{{g}}}_{\text {N}}^{(r)}=\dfrac{g_{\text {N}}(x_3)}{\left| g_{\text {N}}^{0}(x_3)\right| }=\dfrac{(g_{\text {N}})_3}{H^{(r)}}= \dfrac{2\tau }{H^{(r)}}\left( {{\Lambda }_{\text {N}}^{(r)}-t_{\text {N}}} \right) , \end{aligned}$$

108 $$\begin{aligned}&{\bar{{g}}}_{\text {T}}^{(r)}=\dfrac{g_{\text {T}}(x_3)}{\left| g_{\text {T}}^{0}(x_3)\right| }=\dfrac{(g_{\text {T}})_3}{H^{(r)}}= \dfrac{2\tau }{H^{(r)}}\left( {{\Lambda }_{\text {T}}^{(r)}-t_{\text {T}}} \right) . \end{aligned}$$

Substituting (107, 108) in (97, 98) we obtain

109 $$\begin{aligned} {\mathbf {{R}}}_{\text {RBM}}({\mathbf {{d}}},\pmb {\Lambda })&={\mathbf {{R}}}_{\text {RBM},N}({\mathbf {{d}}},\Lambda _{\text {N}})+{\mathbf {{R}}}_{\text {RBM},T}({\mathbf {{d}}},\pmb {\Lambda }), \end{aligned}$$

110 $$\begin{aligned} {\mathbf {{R}}}_{\text {RBM},N}({\mathbf {{d}}},\Lambda _{\text {N}})&=\sum _{r=1}^{N_{\text {r}}}c\int _{\Gamma _{\text {D}}^{(r)}}\left( {{\bar{{g}}}_{\text {N}}({\mathbf {{d}}},{\Lambda }_{\text {N}}){\mathbf {{n}} \left( {\sum _{a}{\pmb {\nabla }}({\mathbb {N}}_{a})} \right) \cdot {\mathbf {{n}}}}} \right) \nonumber \\&\qquad {\bar{{g}}}_{\text {N}}({\mathbf {{d}}},{\Lambda }_{\text {N}})\;d\Gamma , \end{aligned}$$

111 $$\begin{aligned} {\mathbf {{R}}}_{\text {RBM},T}({\mathbf {{d}}},\pmb {\Lambda })&=\sum _{r=1}^{N_{\text {r}}}c\int _{\Gamma _{\text {D}}^{(r)}} \left\{ {\bar{{g}}}_{\text {N}}({\mathbf {{d}}},{\Lambda }_{\text {N}}){\mathbf {{n}} \left( {\sum _{a}{\pmb {\nabla }}({\mathbb {N}}_{a})} \right) \cdot {\mathbf {{t}}}}\right. \nonumber \\&\quad \left. +\; {\bar{{g}}}_{\text {N}}({\mathbf {{d}}},{\Lambda }_{\text {N}}){\mathbf {{t}} \left( {\sum _{a}{\pmb {\nabla }}({\mathbb {N}}_{a})} \right) \cdot {\mathbf {{n}}}}\right. \end{aligned}$$

112 $$\begin{aligned}&\quad \left. +\;{\bar{{g}}}_{\text {T}}({\mathbf {{d}}},{\Lambda }_{\text {T}}){\mathbf {{t}} \left( {\sum _{a}{\pmb {\nabla }}({\mathbb {N}}_{a})} \right) \cdot {\mathbf {{t}}}}\right\} {\bar{{g}}}_{\text {T}}({\mathbf {{d}}},{\Lambda }_{\text {T}})\;d\Gamma , \end{aligned}$$

with

113 $$\begin{aligned}&{\bar{{g}}}_{\text {N}}^{(r)}({\mathbf {{d}}},{\Lambda }_{\text {N}})= \dfrac{2\tau }{H}^{(r)}\left( {{\Lambda }_{\text {N}}^{(r)}-{\mathbf {{n}}}\cdot {\mathbf {{P}}}_{\text {I}}(\mathbf {{d}})\cdot \mathbf {{N}}} \right) , \end{aligned}$$

114 $$\begin{aligned}&{\bar{{g}}}_{\text {T}}^{(r)}({\mathbf {{d}}},{\Lambda }_{\text {T}})= \dfrac{2\tau }{H}^{(r)}\left( {{\Lambda }_{\text {T}}^{(r)}-{\mathbf {{t}}}\cdot {\mathbf {{P}}}_{\text {I}}(\mathbf {{d}})\cdot \mathbf {{N}}} \right) . \end{aligned}$$

Note that the modifications to the mechanical residual of those domains that exhibit RBMs $\Omega ^{(m)}$ are performed involving nodal displacements ${\mathbf {{d}}}^{(m)}$ of the same domain and Lagrange multipliers defined at the interface patch $D^{(r)}$ assuming that only those interface patches $D^{(r)}$ adjacent to $\Omega ^{(m)}$ are selected (cf. Fig. 6). The new submatrices of the global dual system (70) corresponding to domains with rigid body modes $\Omega ^{(m)}$ can be defined as

115 $$\begin{aligned}&\left[ \begin{array}{l@{\quad }l} {\mathbf {{K}}}_{\text {dd}}^{(m)} &{} {\mathbf {{K}}}_{\text {d}\Lambda }^{(m)} \\ {\mathbf {{K}}}_{\Lambda \text {d}}^{(m)} &{} {\mathbf {{K}}}_{\Lambda \Lambda } \\ \end{array}\right] = \left[ \begin{array}{l@{\quad }l} \dfrac{\partial \left( {{\tilde{\mathbf {{R}}}}_{\text {mech}}({\mathbf {{d}}},{\pmb {\Lambda }})} \right) ^{(m)}}{\partial {\mathbf {{d}}}^{(m)}} &{} \dfrac{\partial \left( {{\tilde{\mathbf {{R}}}}_{\text {mech}}({\mathbf {{d}}},{\pmb {\Lambda }})} \right) ^{(m)}}{\partial {\pmb {\Lambda }}^{(m)}}\\ \dfrac{\partial \left( {{\tilde{\mathbf {{R}}}}_{\lambda }({\mathbf {{d}}},{\pmb {\Lambda }})} \right) ^{(m)}}{\partial {\mathbf {{d}}}^{(m)}} &{} \dfrac{\partial {\tilde{\mathbf {{R}}}}_{\lambda }({\mathbf {{d}}},{\pmb {\Lambda }})}{\partial {\pmb {\Lambda }}} \end{array}\right] , \end{aligned}$$

116 $$\begin{aligned}&\left[ \begin{array}{l} {\mathbf {{r}}}_{\text {d}}^{(m)} \\ {\mathbf {{r}}}_{\Lambda } \\ \end{array}\right] = \left[ \begin{array}{l} \left( {\tilde{{\mathbf {{R}}}}_{\text {mech}}({\mathbf {{d}}},{\pmb {\Lambda }})} \right) ^{(m)}\\ {\tilde{\mathbf {{R}}}}_{\lambda }({\mathbf {{d}}},{\pmb {\Lambda }}) \end{array}\right] , \end{aligned}$$

where

117 $$\begin{aligned} \left( {\tilde{{\mathbf {{R}}}}_{\text {mech}}({\mathbf {{d}}},{\pmb {\Lambda }})} \right) ^{(m)}=\left( {{{\mathbf {{R}}}}_{\text {mech}}({\mathbf {{d}}},{\pmb {\Lambda }})} \right) ^{(m)}+ \left( {{\mathbf {{R}}}_{\text {RBM}}({\mathbf {{d}}},\pmb {\Lambda })} \right) ^{(m)}. \end{aligned}$$

Consequently, the bandwidth in (70) is kept also for the contributions to domains with RBMs and, for this reason, the system can be processed in a parallel fashion as detailed in (78–80).

#### *Remark 2.10*

Equations (113, 114) are obtained assuming linear (constant strain) interface patches as adopted in the examples presented in this work. If a higher order interpolation is employed at the interface patches, a mean value of the normal and tangential traction $t_{\text {N}}$ and $t_{\text {T}}$ can be used along $\Gamma _{\text {D}}^{(p)}$ such that the relations (113, 114) can still be utilized.

#### *Remark 2.11*

In all our computations we considered all interface patches $D^{(r)}$ adjacent to domain $\Omega ^{(m)}$. If the base-line of the patch $D^{(r)}$ chosen to avoid the RBM is not located at $\partial \Omega ^{(m)}$, contributions to adjacent domains would be expected in (70) and, therefore, the system could not be properly processed in a parallel fashion.


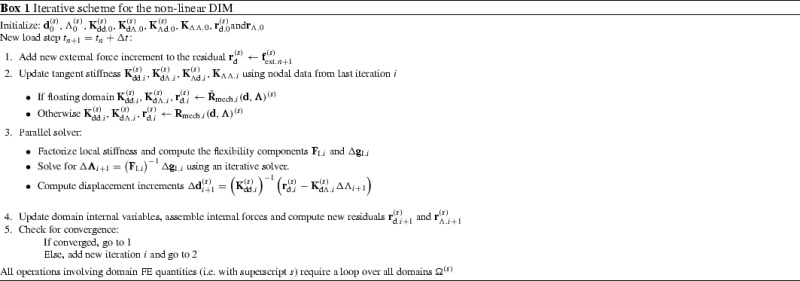


### Iterative scheme for the non-linear DIM

The linearized set of equations in (70) obtained with a Newton-like scheme is solved iteratively for each load/time step $\Delta t$ as done in the so-called Newton-Krylov-Schur methods [6, 12]. In this view, a first type of iterations refer to the solution of the non-linear problem with successive linear approximations. A second type of iterations arise from the solution of the flexibility problem in (77) where usually Conjugate Gradient or GMRES iterates are considered. Finally, the Schur complements are utilized for the local solutions at each domain $\Omega ^{(s)}$ (80).

Assuming a fix domain decomposition and a given Delaunay interface discretization, the iterative scheme for the non-linear DIM framework is summarized in Box 1.

**Fig. 7:**
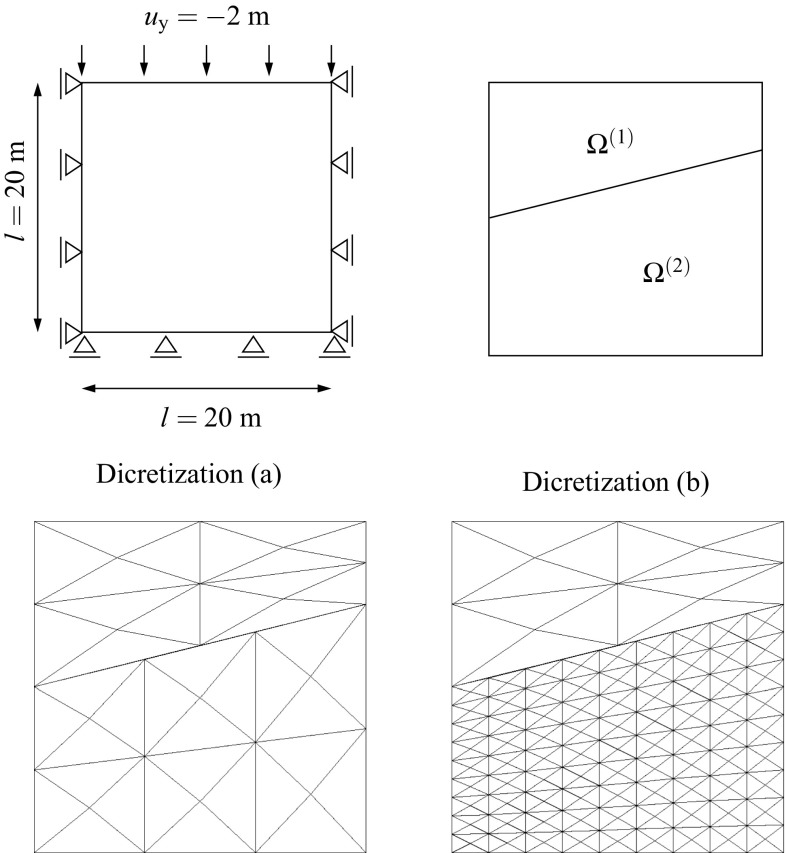
Geometry, boundary conditions and domain decomposition (*top*). Domain discretizations using linear T3 elements (*bottom*)

**Fig. 8:**
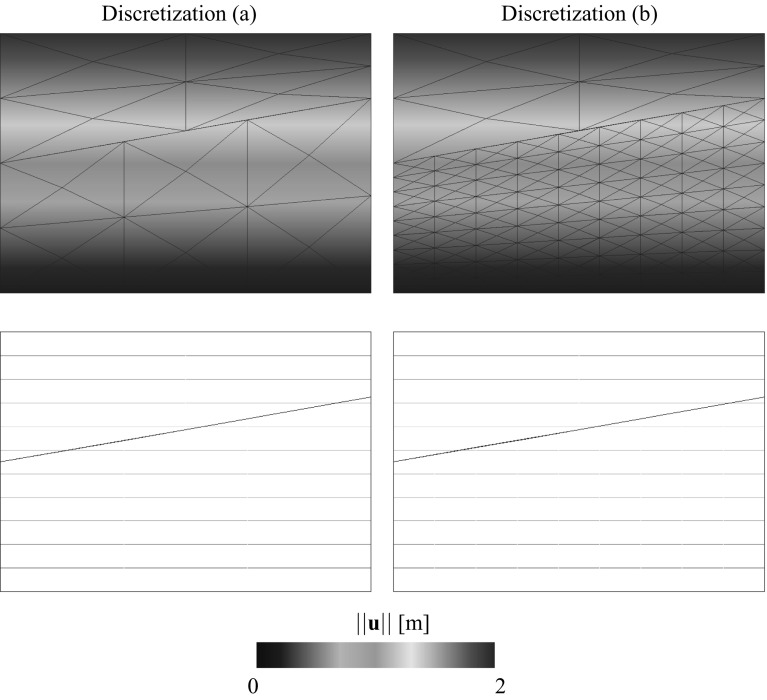
Total displacement $\left| \left| {\mathbf {{u}}}\right| \right|$ contours (*top*) and contour lines (*bottom*) within the deformed configurations for discretizations (**a**) and (**b**). 3 $\times$ displacement magnification

## Framework validation through representative simulations

In the following we present a number of academic examples which highlight the accuracy and convergence properties of the framework. Attention is focused on the continuity of the solution at the interface, the convergence rate upon mesh refinement and a qualitative comparison of the advantages and eventual pitfalls against existing formulations. Infinitesimal strain theory is utilized in all examples except from the one reported in Sect. 3.5 where finite strain theory is considered. Additionally, the stabilization parameter $\tau$ (cf. Eqs. 63–68) is set to $10^{-7}$ in all our computations except from the results reported by the end of Sect. 3.4 where a sensitivity analysis is performed varying the values of $\tau$.

### Patch test

The so-called ‘patch test’ is specially selected to verify the correct transference of information throughout the interface. A compression analysis is performed on a two-dimensional homogeneous quadrilateral specimen. Plane strain conditions are assumed and a linear elastic constitutive law is considered with Young’s modulus $E=2.1 \times 10^{2}$ MPa and Poisson’s ratio $\nu =0.3$. The geometry, boundary conditions and domain discretizations are depicted in Fig. 7.

The quadrilateral specimen is submitted to an homogeneous strain state where

118 $$\begin{aligned} {\pmb {\varepsilon }}=\left[ \begin{array}{l@{\quad }l@{\quad }l} 0 &{} 0 &{} 0 \\ 0 &{} \dfrac{u_{\text {y}}}{l} &{} 0 \\ 0 &{} 0 &{} 0 \\ \end{array}\right] . \end{aligned}$$

Considering the linear elastic constitutive relation

119 $$\begin{aligned} {\pmb {\sigma }}=\lambda Tr({\pmb {\varepsilon }}){\mathbf {{1}}} + 2\mu {\pmb {\varepsilon }} \end{aligned}$$

with Lamé constants

120 $$\begin{aligned} \lambda =\dfrac{\nu E}{(1+\nu )(1-2\nu )},\quad \mu =\dfrac{E}{2(1+\nu )} \end{aligned}$$

and ${\mathbf {{1}}}$ being the second order unity tensor, the analytical solution reads $\sigma _{\text {x}}=-1.2115\times 10^{4}$ Pa and $\sigma _{\text {y}}=-2.8269\times 10^{4}$ Pa. These values are obviously constant throughout the specimen, and are taken as the reference solution with nine significant decimal digits when compared to the stress distributions obtained through our simulations using double precision floating points.

Displacement contours for both discretizations are shown in Fig. 8 within the corresponding deformed configurations. Contour lines show that continuity is satisfied throughout the whole specimen at this observation scale. The horizontal and vertical stress fields are constant and identical for both cases according to the machine precision and therefore not reported. However the relative error $e_\text {r}$ between the numerical and reference stresses is $1.2\times 10^{-8}$ for the horizontal stress and $1.1\times 10^{-8}$ for the vertical stress.

Since the maximum relative error is order $10^{-8}$, it is concluded that the proposed methodology passes the patch test and provides an adequate transference of information across a non-conforming interface.

### Patch test with floating subdomains

**Fig. 9:**
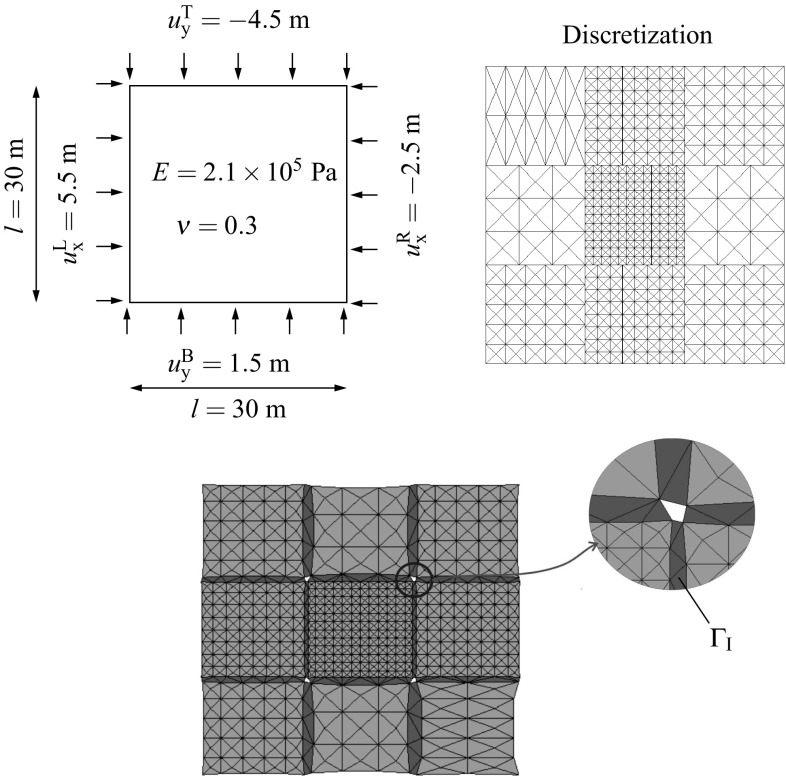
Geometry, boundary conditions and material parameters (*top left*). Domain discretizations using linear T3 elements (*top right*). Detail of the interface discretization $\Gamma _{\text {I}}$ at corner points (*bottom*)

In the same spirit as the previous example a patch test is set up using a domain discretization containing one floating domain. The objective is, therefore, to verify that the algorithm to handle floating domains developed in Sect. 2.5 does not affect the resulting solution. To this end, a biaxial compression test is imposed to a quadrilateral specimen divided into nine domains as shown in Fig. 9 and a linear elastic constitutive law is considered with plane strain conditions. The parameter *c* used to overcome the appearance of RBMs (cf. 93–98) is set to $10^{-4}$ in all examples that require the treatment of floating domains.

**Fig. 10:**
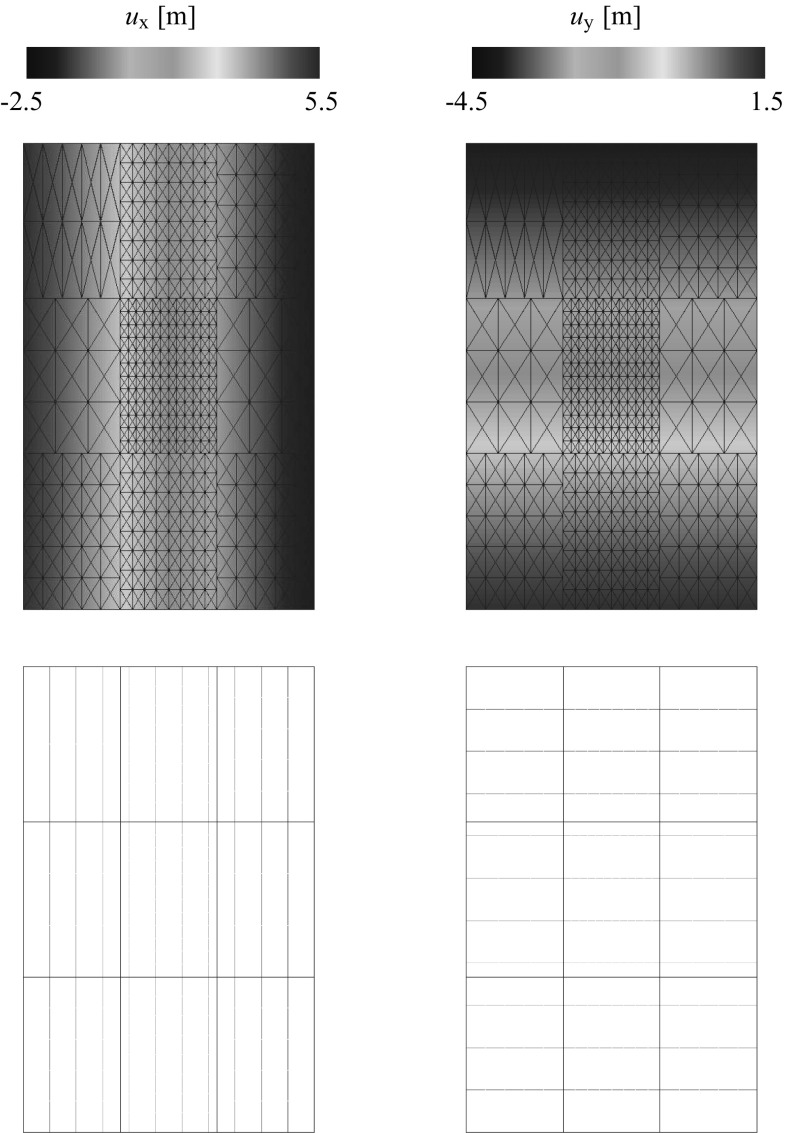
Horizontal (*left*) and vertical (*right*) displacement contours (*top*) and contour lines (*bottom*) within the deformed configurations. 1.75 $\times$ displacement magnification

**Fig. 11:**
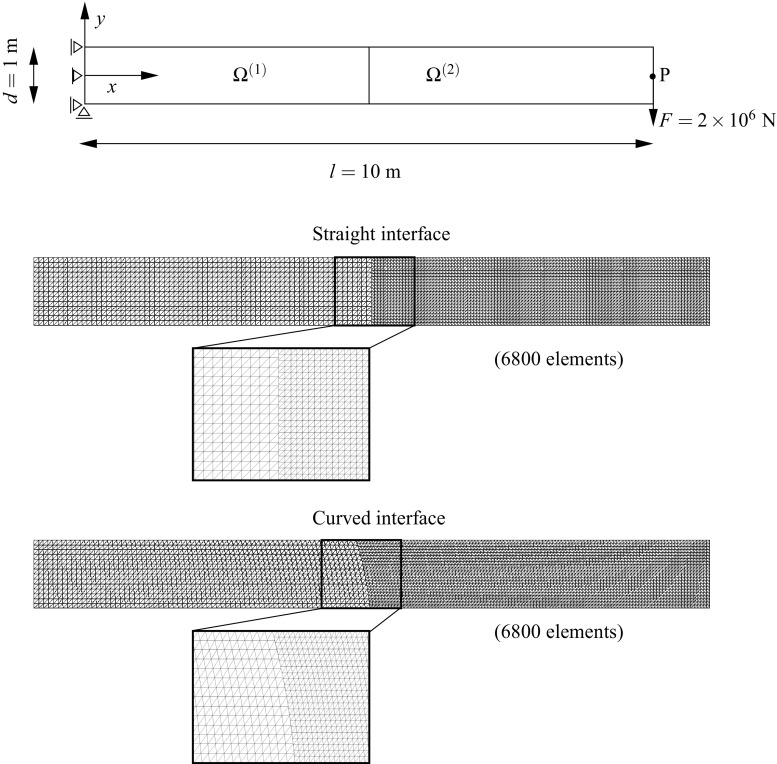
Boundary conditions and domain discretizations for the cantilever beam test

Note that interface corner points are correctly connected using the interface discretization. The boundary conditions result in an homogeneous strain state where

121 $$\begin{aligned} {\pmb {\varepsilon }}=\left[ \begin{array}{l@{\quad }l@{\quad }l} \dfrac{\delta _{\text {x}}}{l} &{} 0 &{} 0 \\ 0 &{} \dfrac{\delta _{\text {y}}}{l} &{} 0 \\ 0 &{} 0 &{} 0 \\ \end{array}\right] \end{aligned}$$

with $\delta _{\text {x}}=u^{\text {R}}_{\text {x}}-u^{\text {L}}_{\text {x}}$ and $\delta _{\text {y}}=u^{\text {T}}_{\text {y}}-u^{\text {B}}_{\text {y}}$. Considering the linear elastic constitutive relation and Lamé constants in (119, 120), respectively, the analytical stresses read $\sigma _{\text {x}}=-9.9615\times 10^{4}$ Pa and $\sigma _{\text {y}}=-8.8846\times 10^{4}$ Pa.

Displacement contour lines in the deformed configuration plotted in Fig. 10 show that displacement continuity is again satisfied throughout the whole specimen at this observation scale. The obtained horizontal and vertical stress fields are obviously constant and the relative error $e_\text {r}$ between the numerical and reference stresses is $1.5\times 10^{-9}$ for the horizontal stress and $1.8\times 10^{-9}$ for the vertical stress.

**Fig. 12:**
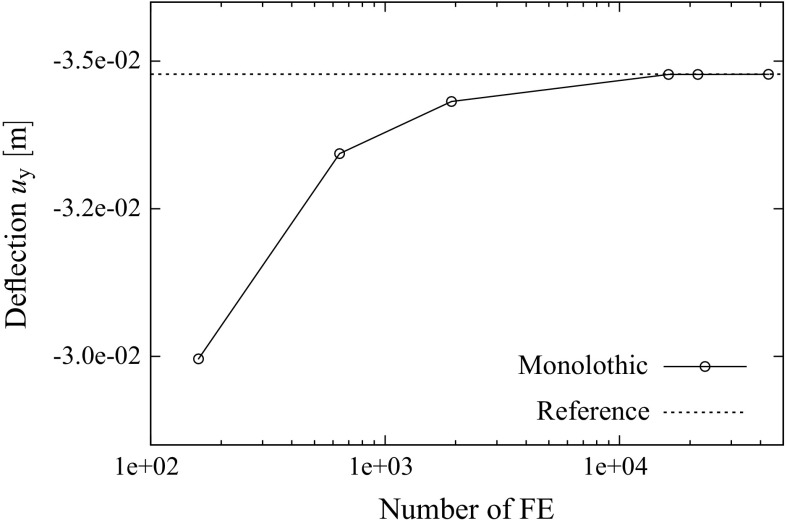
Mesh sensitivity analysis with a monolithic approach

Both relative errors are significantly small and, therefore, it is concluded that the patch test is successfully passed in those cases where floating subdomains are present. This indicates that the proposed non-intrusive methodology to handle floating domains does not affect the accuracy of the domain decomposition framework.

### Cantilever beam test

The following example is based on a test proposed by Herry et al. [24] where the deflection at one end of a cantilever beam is measured in order to assess the performance of the domain decomposition method when splitting the beam into two domains connected at a non-conforming interface. The material is linear elastic with Young’s modulus $E=2.1\times 10^{11}\,\mathrm{N/m}^2$ and Poisson’s ratio $\nu =0.3$. The two-dimensional test is conducted under plane strain conditions and the geometry, boundary conditions and domain discretizations are shown in Fig. 11.

**Fig. 13:**
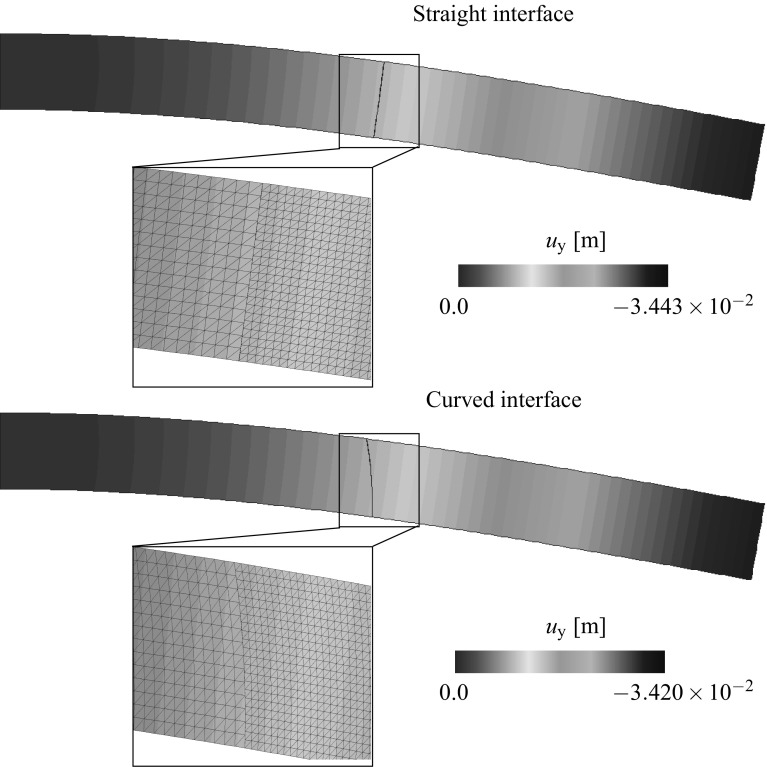
Vertical displacement contours employing straight and curved interfaces. 35 $\times$ displacement magnification

The reference solution for the right end deflection is obtained through simulations with a monolithic approach, i.e. considering a single discretization for the whole specimen and employing a standard FE approach. A mesh sensitivity analysis is performed (cf. Fig. 12) and the reference deflection $u^{\text {ref}}_{\text {y}}=-3.478\times 10^{-2}$ m is selected which corresponds to a mesh discretization similar to the one chosen in the domain decomposition (DD) approach.

The vertical displacement contours for both straight and curved interfaces are shown in Fig. 13. Note that displacement continuity is fulfilled across the straight and curved interfaces.

The high accuracy of the DD approach compared to the reference monolithic solution is proven in Table 1. Both decompositions provide accuracy percentages which are above $98\,\%$.

**Table 1:** Cantilever beam test. Accuracy of the proposed approach compared to the reference numerical solution

	Straight interface	Curved interface
Number of $\Lambda$	36	36
$\displaystyle \dfrac{u_{\text {y}}}{u^{\text {ref}}_{\text {y}}}$	99.00 %	98.33 %

**Table 2:** Cantilever beam test. Accuracy of similar approaches compared to a reference theoretical solution

	Coarse mortar mesh	Fine mortar mesh	Dual DD method
Number of $\Lambda$	15	23	35
$\displaystyle \dfrac{u_{\text {y}}}{u^{\text {ref}}_{\text {y}}}$	79.06 %	99.86 %	99.84 %

**Fig. 14:**
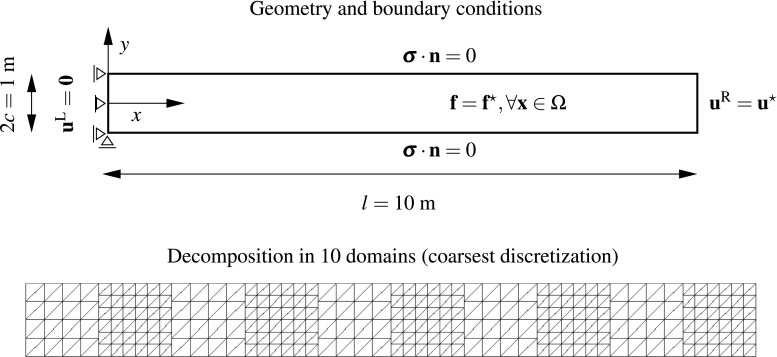
Boundary conditions (*top*) and domain decomposition using the coarsest FE discretization (*bottom*)

A similar example was carried out by Herry et al. [24] where the cantilever beam was analyzed using bilinear quadrilateral FE. Table 2 is reproduced from this study and compares the performance between mortar methods and a dual domain decomposition approach developed for non-matching meshes. It is worth noting that the accuracy of the deflection provided in the present contribution is very much comparable to the accuracies of the above mentioned approaches. It is noticeable in Table 2 that the original mortar method performs poorly when the coarse mesh is used to define the mortar surface. This is obviously regarded as a considerable drawback of the method since in a general case the distinction between coarse and fine discretizations at the interface might not be straightforward. The approach based on dual domain decomposition methods does not suffer from this shortcoming since a third surface is constructed with a particular arrangement of Lagrange multipliers that leads to an optimum matching condition regarding the kinematic continuity. However, this approach is based on the assumption that the three surfaces have the same geometry. This implies that, upon discretization, a number of nodes, e.g. extreme nodes, are common. This assumption is reasonable for cases in which the domains are originated from the decomposition of a continuous body. Conversely, if the domains are glued at a common surface and discretized independently, this condition might not be realistic.

More advanced mortar methods employed nowadays [40–42, 50] consider a carefully chosen Lagrange multiplier space based on stability and optimality considerations or even a third auxiliary surface with an optimal node collocation to minimize the error at the interface integrals. This may not lead to the suboptimal performance shown in Table 2 but, for the case of a third auxiliary surface, extra degrees of freedom (possibly condensible) are required and, therefore, the cost and complexity of the formulation is increased. For the case of an heterogeneous interface, the performance in terms of accuracy of such advanced mortar methods would not be affected. However, the DIM method outlined in this contribution for two-dimensional applications distinguishes from these techniques in the sense that no extra projection surfaces and extra DOFs are required for a general geometrically incompatible interface since this is automatically taken into account by the interface mesh. It is not the author’s intention to provide a review study of all recent mortar variants but rather compare the accuracy of the DIM method with situations in which original mortar methods would perform optimally. We believe that more advanced mortar technologies would provide a comparable accuracy to the one observed by original mortar techniques when the finest mesh is selected as the mortar surface. In such scenarios, the DIM method would be considered a computationally cheaper alternative.

### Convergence analysis and dependence on the stabilization parameter $\tau$

The effect of mesh refinement at the interface is studied in this section. Based on the work of Girault et al. [18] a test is set up in a two dimensional cantilever beam under plane strain conditions. Infinitesimal strain theory is utilized and the material follows a linear elastic constitutive law with Young’s modulus $E=2.0\times 10^5\,\mathrm{N/m}^2$ and Poisson’s ratio $\nu =0.3$.

**Fig. 15:**
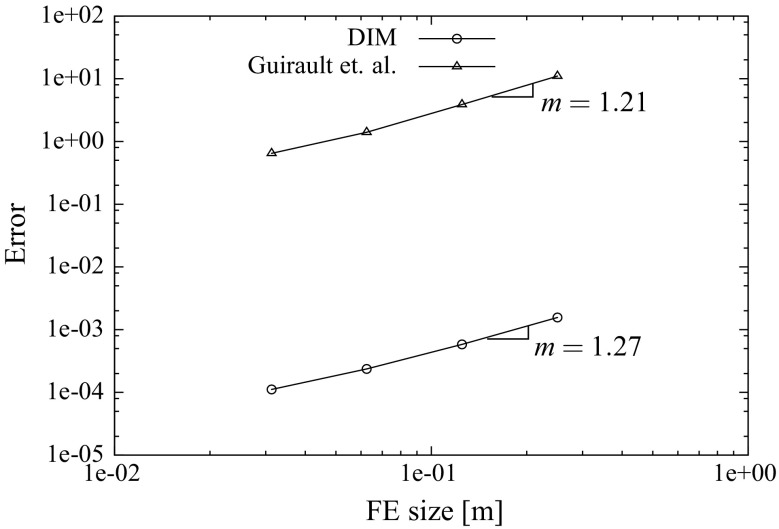
Local convergence error based on the displacement jump norm $\left| \left| \llbracket \mathbf{u}\rrbracket \right| \right| _{\text {L}_2}$ in (124) for different mesh discretizations

**Fig. 16:**
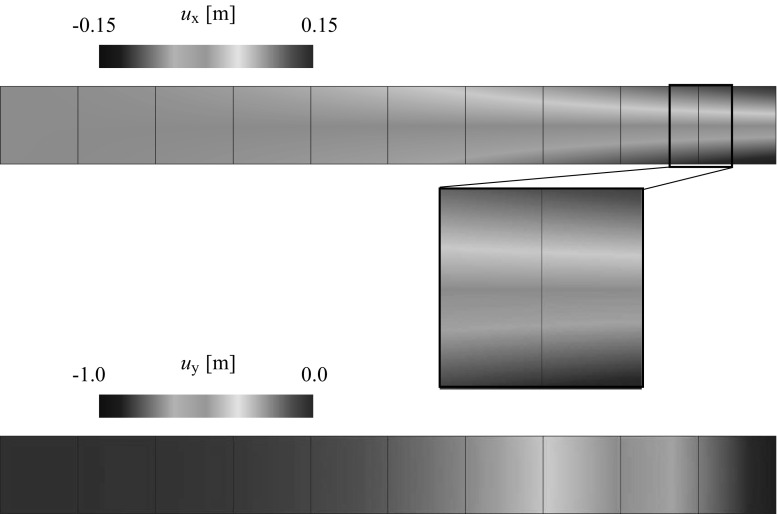
Horizontal (*top*) and vertical (*bottom*) displacement contours for the finest FE discretization

As sketched in Fig. 14, homogeneous displacement boundary conditions are imposed at the left end of the beam ${\mathbf {{u}}}^{\text {L}}={\mathbf {{0}}}$ and the right end follows the displacement ${\mathbf {{u}}}^{\text {R}}={\mathbf {{u}}}^{\star }$ where

122 $$\begin{aligned} {\mathbf {{u}}}^{\star }=\left[ \begin{array}{l} -3\alpha x^2 y \\ \alpha x^3 + \dfrac{3\alpha \lambda }{\lambda + 2 \mu }x(y^2-c^2)\\ \end{array}\right] , \end{aligned}$$

$\lambda$ and $\mu$ being the Lamé constants described in (120) and $\alpha =-1.0\times 10^{-3} \mathrm{m}^{-2}$. The body force field on the beam reads

123 $$\begin{aligned} {\mathbf {{f}}}^{\star }=\left[ \begin{array}{l} 6 \alpha \mu \dfrac{3 \lambda + 4 \mu }{\lambda + 2 \mu }y\\ 0 \\ \end{array}\right] . \end{aligned}$$

This condition is translated into homogeneous tractions ${\pmb {\sigma }} \cdot {\mathbf {{n}}}=0$ at the top and bottom surfaces of the beam as indicated in Fig. 14.

The beam is split into ten domains as indicated in Fig. 14 and an alternate regular discretization is considered between domains such that all interfaces are non-conforming. Four levels of refinement are employed which correspond to element sizes ranging from 1 / 4 to 1 / 32 m for one set of domains and 1 / 6 to 1 / 48 m for the alternate set of domains.

The error is measured taking into account the displacement jump $\llbracket \mathbf{u}\rrbracket$ at the interface $\Gamma _{i,j}$ between domains $\Omega _i$ and $\Omega _j$ as

124 $$\begin{aligned} {\left| \left| \llbracket \mathbf{u}\rrbracket \right| \right| _{\text {L}_2}=\displaystyle \left( {\int _{\Gamma _{i,j}}\left| {\mathbf {{u}}}_i-P_{\Gamma _i}({\mathbf {{u}}}_j)\right| ^2 \; d\Gamma _{i,j}} \right) ^{1/2}, \,\,\,\,\forall \Gamma _{i,j} \in \Gamma _{\text {I}},} \end{aligned}$$

where $P_{\Gamma _i}({\mathbf {{u}}}_j)$ denotes the projection of the coarse domain interface displacements $u_j$ onto the fine interface discretization $\Gamma _i$. It can be observed that the error $\left| \left| \llbracket \mathbf{u}\rrbracket \right| \right| _{\text {L}_2}$ reduces with decreasing element size and the rate of change *m* is about 1.27 between the first two discretizations (cf. Fig. 15). This is in accordance with the results presented by Girault et al. [18] where a different error is computed which takes into account the prescribed boundary and Lagrange multipliers at the interface.

Additionally, the displacement norm $\left| \left| {\mathbf{u}}-{\mathbf {{u}}}_{\text {h}}\right| \right| _{\text {L}_2}$ is calculated using the approximated ${\mathbf{u}}_{\text {h}}$ and theoretical solution ${\mathbf{u}}$ throughout the specimen as:

125 $$\begin{aligned} {\left| \left| {\mathbf{u}}-{\mathbf {{u}}}_{\text {h}}\right| \right| _{\text {L}_2}=\displaystyle \left( {\int _{\Omega }\left| {\mathbf {{u}}}-{\mathbf {{u}}}_{\text {h}}\right| ^2\; d\Omega } \right) ^{1/2}.} \end{aligned}$$

The convergence study summarized in Fig. 15 shows satisfactory results when the framework is compared to similar techniques. Displacement jumps at the interfaces are hardly visible at the displacement contour plots given in Fig. 16 which reveals that displacement continuity is sufficiently satisfied in the present approach.

**Fig. 17:**
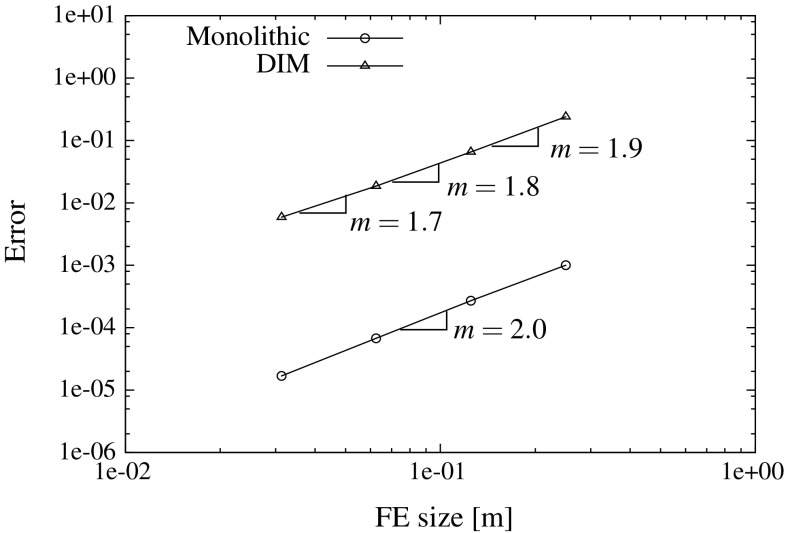
Global convergence error based on the displacement norm $\left| \left| {\mathbf{u}}-{\mathbf {{u}}}_{\text {h}}\right| \right| _{\text {L}_2}$ in (125) for different mesh discretizations

**Fig. 18:**
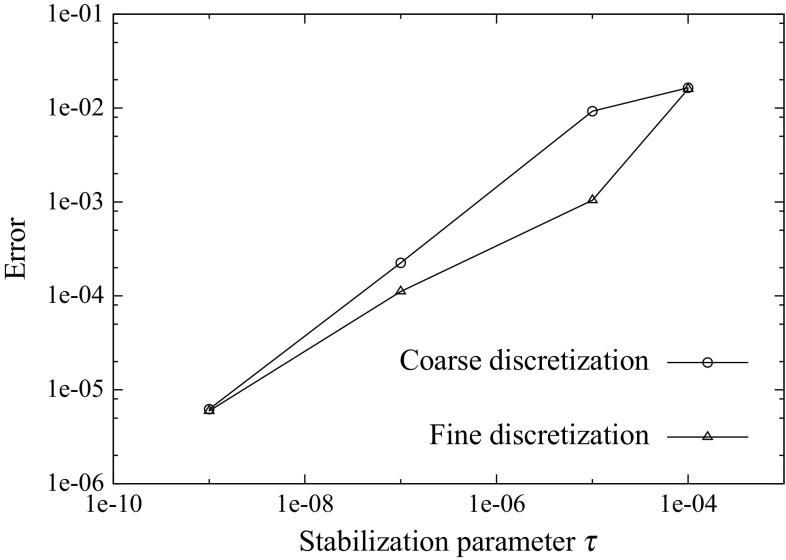
Error $\left| \left| \llbracket \mathbf{u}\rrbracket \right| \right| _{\text {L}_2}$ for different values of the stabilization parameter $\tau$

**Fig. 19:**
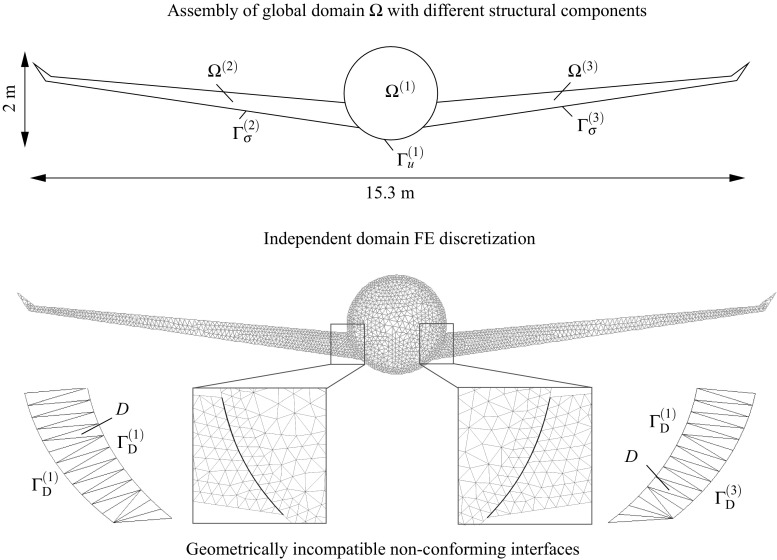
Plane structure assembly. Geometry (*top*) and domain decomposition (*bottom*)

Results in Fig. 17 show the expected convergence order $\mathcal {O}(h^2)$ using the displacement norm in (125) for a monolithic analysis. When the analysis is performed using the proposed domain decomposition approach using different mesh refinements and non-conforming interfaces, the obtained convergence order is found lower than $\mathcal {O}(h^2)$. The reason for this suboptimal behaviour is related with the use of piece-wise constant Lagrange multipliers at each interface patch. Although this choice has a positive impact on the simplicity of the framework implementation, the exact theoretical convergence order may not be recovered. A more thorough study considering higher interpolations of the Lagrange multiplier field and its impact on the presented framework is considered a topic for future research.

**Fig. 20:**
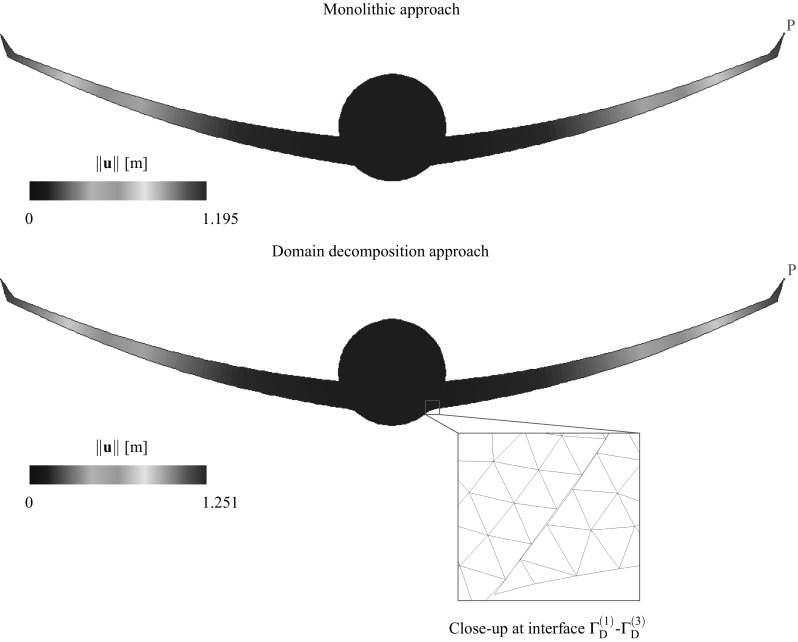
Total displacement contours of the monolithic (*top*) and domain decomposition approaches (*bottom*) in the deformed configurations

The sensitivity of the analysis with respect to the stabilization parameter $\tau$ is reported in Fig. 18 where the jump error $\left| \left| \llbracket \mathbf{u}\rrbracket \right| \right| _{\text {L}_2}$ is plotted against different values of $\tau$ for the coarsest and finest discretization sets. It is observed that the error diminishes with decreasing values of $\tau$. Additionally, one can utilize remarkably low values of the stabilization parameter upon mesh refinement which confirms the consistency character indicated in Sect. 2.3. In general, coarser meshes tend to give higher error values for a given value of the stabilization parameter $\tau$.

**Table 3:** Deflections at point P using the monolithic and domain decomposition approaches

	Monolithic	Domain decomposition	Relative error
	$\left| \left| {\mathbf {{u}}}_{\text {M}}\right| \right|$ [m]	$\left| \left| {\mathbf {{u}}}_{\text {DDM}}\right| \right|$ [m]	$e_{\text {r}}=\dfrac{\left| \left| {\mathbf {{u}}}_{\text {M}}\right| \right| -\left| \left| {\mathbf {{u}}}_{\text {DDM}}\right| \right| }{\left| \left| {\mathbf {{u}}}_{\text {DDM}}\right| \right| }$
Point P	1.195	1.251	$4.676\,\%$

### Geometrically incompatible non-matching meshes

The following example consists in the assembly of a simplified plane-like structure where two geometrically incompatible interfaces are identified which connect the wings to the plane fuselage (cf. Fig. 19). A distributed force of 0.5 N/m is applied at the bottom part of both wings to emulate the lift experienced during flight conditions. Several points at the fuselage section are imposed null Dirichlet boundary conditions in order to restrict the global rigid body modes. The material is considered hyperelastic [48] satisfying

126 $$\begin{aligned} {\mathbf {{S}}}=\lambda tr({\mathbf {{E}}}){\mathbf {{1}}}+2\mu {\mathbf {{E}}}, \end{aligned}$$

${\mathbf {{S}}}$ being the second Piola–Kirchhoff stress tensor, ${\mathbf {{E}}}$ the Green–Lagrange strain, and $\lambda$ and $\mu$ the Lamé constants. Due to the simplicity of the model, i.e. discretized by solid elements and not thin-shells, the material parameters are chosen arbitrarily and are not sought to mimic a real situation but rather illustrate a target case for the use of geometrically incompatible non-conforming interfaces. In the current simulations the Young’s modulus and Poisson’s ratio are set to $2.0\times 10^4$ MPa and 0.3, respectively.

In order to assess the performance of the DIM in this example, the vertical displacement of the wing end (point P) is monitored and compared to the one obtained with a reference monolithic approach considering a similar spatial discretization. As it can be observed in the contour plots in Fig. 20 and the deflections reported in Table 3, the results for the geometrically incompatible non-conforming interface are remarkably close to the ones obtained with a monolithic approach used as the reference solution for this problem. Note that the interface gap depicted in the close-up in Fig. 20 is hardly visible and it can be concluded that the methodology shows satisfactory results and proves to be remarkably competent against the most demanding cases.

## Conclusions and future perspectives

The kernel of the proposed DIM to be used in domain decomposition methods and presented in this contribution resides in an explicit discretization of the interface by means of a zero-thickness Delaunay triangulation. This is accomplished through a fictitious contraction of the subdomains at the interface which allows for a proper discretization between the shrunk domain boundaries. The fictitious contraction has no impact on the solution of the problem since all calculations are performed using the original coordinates. Moreover, it is shown that the integrals over the zero-thickness interface are bounded despite the fact that the integrand is not bounded. The method is grounded in the so called Nitsche methods in which a stabilization term is added at the constraint equations. In this manner, zero diagonal terms are not present at the global system and instabilities are avoided if the LBB condition is not fulfilled by the chosen discretization. This process is viewed as a consistent penalty method since the stabilization term vanishes with progressive mesh refinement and the penalty factor can be made significantly small without affecting the results.

The methodology is inherited from the field of contact mechanics and, for this reason, is regarded as more general than existing domain decomposition strategies since there is no need for a fixed interface geometry that needs to be shared by the decomposed domains at both sides of the interface. This is for instance the case of some dual domain decomposition techniques in which the limit DOFs at the interface need to be common in both adjacent domain discretizations. Moreover, the generation of interface patches is independent of the choice of slave and master sides in contrast with early mortar methods and, for this reason, the methodology is regarded less prone to errors related with such choice. More evolved mortar methodologies are able to automatically handle these situations by considering, for instance, an extra interface surface from which a particular distribution of DOFs serves to construct an optimal set of interface constraints between adjacent meshes. However, such an intermediate surface involves calculations over extra DOFs which could increase the computational cost and complexity of the approach.

A new non-intrusive strategy to handle floating domains is outlined which adds an extra stabilization term to the energy functional with contributions of all adjacent interface patches. This avoids the calculation of a pseudo-inverse at floating domains and does not destroy the band structure of the global system. For this reason, it does not spoil a possible parallel solution as done with existing dual domain decomposition methods.

The DIM method passes the patch test also for the case of floating subdomains providing continuity of the stress field across the interface which indicates that all new ingredients do not affect the accuracy of the solution when compared to other established techniques. Remarkable continuity of the displacement field across the interface is shown in all reported experiments. A comparable accuracy degree has been observed with independence of the shape of the interfaces. In addition, good convergence rates are reached upon mesh refinement similar to other accurate techniques for non-conforming interfaces although theoretical convergence rates could not be obtained exactly due to the piece-wise constant interpolation of the Lagrange multipliers.

The algorithmic treatment of the subdomains allows for a parallel solution scheme analogous to well established techniques such as dual domain decomposition techniques. In this manner the local factorizations can be tackled by direct solvers and the resulting interface problem can be assembled in a matrix-free fashion and solved with the use of iterative solvers. A full parallel version of the framework involves the construction of adequate preconditioners for the interface problem which was out of the scope of this contribution and it is left as a future research line. In the same spirit, a 3D extension of the method is left as a topic for further research. Preliminary 3D works have been successfully performed for the contact domain case [22] and it is presumed that the technology shows sufficient potential to successfully perform in large 3D cases although challenges are expected concerning a robust 3D Delaunay tetrahedralization for the most complex interface geometries. In any case, the domain decomposition formulation applied to monoscale analysis or ’static’ spatial discretizations has the advantage of performing the interface meshing once at the beginning of the analysis and, therefore, the cost of a new Delaunay tetrahedralization (which could be certainly more involved for a 3D case) is negligible compared to the cost of the whole analysis.

The framework presented in this contribution provides the basis to study complex deformation phenomena involving large strains, e.g. bulk metal forming, and shows a clear potential to tackle multifield applications, e.g. mixed formulations for incompressible and thermo-mechanical problems. In this view, the field of Lagrange Multipliers needs to be extended in order to account for the temperature and pressure fields and it is planned for a future contribution.

The explicit Delaunay triangulation of the interface is expected to positively impact the field of multiresolution problems and adaptive multiresolution analysis. Since arbitrary discretizations can be handled at non-conforming interfaces, adaptive multiresolution analyses can be performed employing an independent ‘on-the-fly’ refinement at particular domains of interest without meshing restrictions. In the same spirit, existing independent discretizations of particular domains can be easily reused and incorporated to the calculations.

Due to its versatility and generality the DIM method is viewed as an attractive alternative to mortar methods and other established dual domain decomposition methods in which the interface geometry and its limits are restricted to the boundary discretization of the connected domains.
